# Discovery of a tsunami deposit from the Bronze Age Santorini eruption at Malia (Crete): impact, chronology, extension

**DOI:** 10.1038/s41598-021-94859-1

**Published:** 2021-07-29

**Authors:** Laurent Lespez, Séverine Lescure, Ségolène Saulnier-Copard, Arthur Glais, Jean-François Berger, Franck Lavigne, Charlotte Pearson, Clément Virmoux, Sylvie Müller Celka, Maia Pomadère

**Affiliations:** 1 Laboratoire de Géographie Physique CNRS, LGP UMR 8591, Meudon, France; 2 Univ Paris Est Creteil, 94010 Créteil, France; 3 Institut Universitaire de France, Paris, France; 4 CNRS, UMR 5600 EVS-IRG, Université de Lyon, Lyon, France; 5 Université Paris 1 Panthéon-Sorbonne, Institut de Géographie, Paris, France; 6 School of Anthropology, University of Arizona, Tucson, AZ 85721 USA; 7 Department of Geosciences, University of Arizona, Tucson, AZ 85721 USA; 8 Archéorient CNRS, Université de Lyon, 69007 Lyon, France; 9 ArScAn CNRS, Univ Paris 1 Panthéon-Sorbonne, 75005 Paris, France

**Keywords:** Natural hazards, Geomorphology

## Abstract

A geomorphological survey immediately west of the Minoan town of Malia (Crete) shows that a tsunami resulting from the Bronze Age Santorini eruption reached the outskirts of the Palatial center. Sediment cores testify a unique erosional event during the Late Minoan period, followed locally by a high energy sand unit comprising marine fauna. This confirms that a tsunami impacted northern Crete and caused an inundation up to 400 m inland at Malia. We obtained a radiocarbon range of 1744–1544 BCE for the secure pre-tsunami context and an interval 1509–1430 BCE for the post-event layer. Examination of tsunami deposits was used to constrain run-up not exceeding 8 m asl. The results open the field for new research on the Bronze Age Santorini tsunami regarding both impact and consequences for the Minoan civilization.

## Introduction

**Figure 1:**
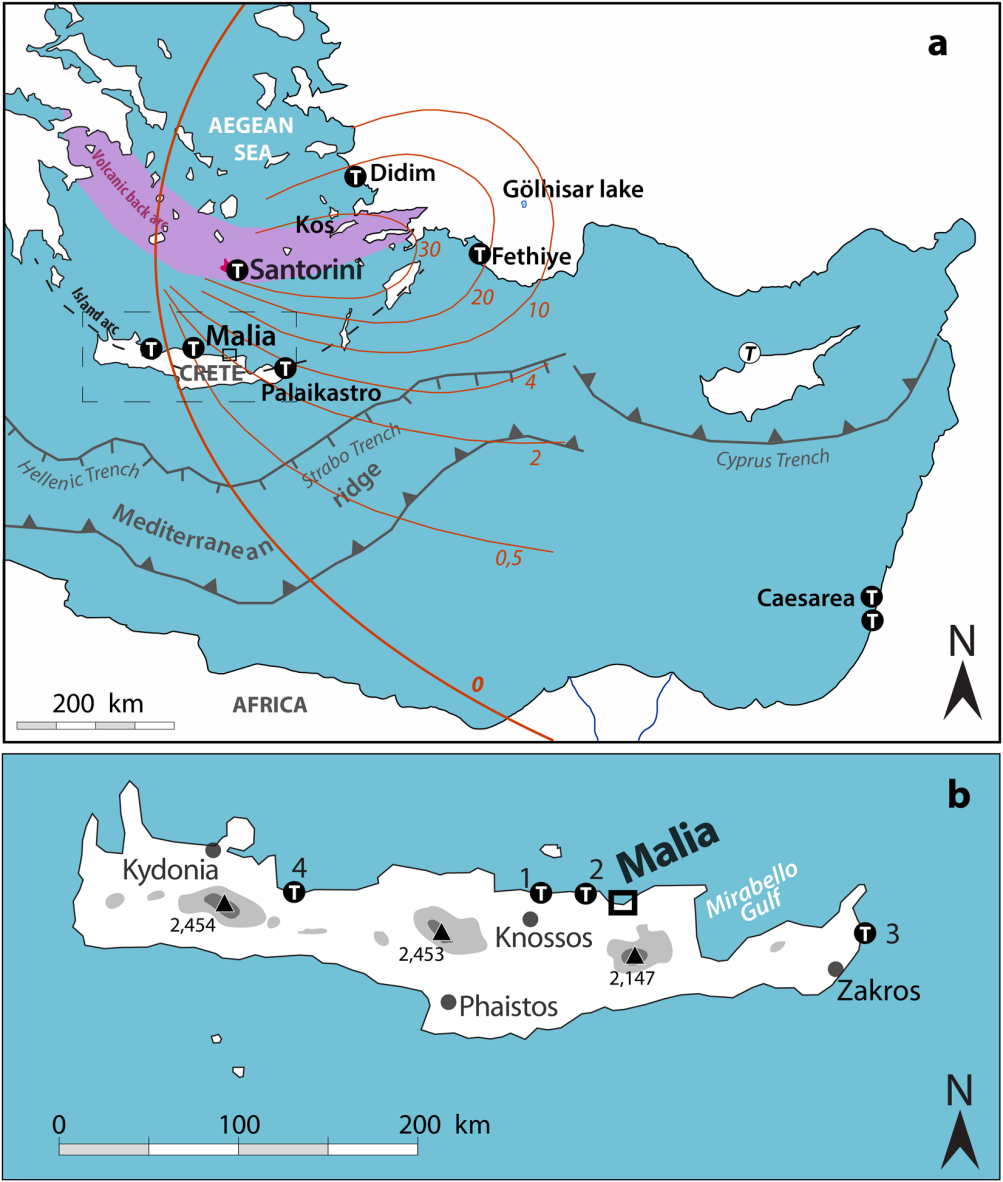
Location of the study area and the Eastern Mediterranean geotectonic setting. (**a**) Red line isopach (in cm) of the ash layer (2); letter T in black circle: putative tsunami deposits. (**b**) Black dots, main centres of the Minoan Crete; White dots, Cretan sites mentioned: 1. Amnisos; 2. Gouves; 3. Palaikastro; 4. Pirgos; Black triangle, main summits, altitude in meters. Edited in Adobe Illustrator CS6 2020 version 16.0.

The palatial town of Malia is one of the major centers of the Minoan civilization (Fig. 1). It flourished during the Middle and, to a lesser extent, Late Bronze Ages, before abandonment in the Late Minoan IIIB (LM IIIB) in the 13th c. BCE^1^. Located on the northern coast of Crete, 120 km south of the Santorini volcano, it provides an opportunity to discuss the consequences of the Bronze Age eruption of Santorini between 1630 and 1525 years BCE. This eruption was one of the most powerful recorded on earth during the last 10,000 years^2,3^ with an estimated Volcanic Explosivity Index (VEI) of 7, and a Dense Rock Equivalent (DRE) of 78–86 km^3^. After minor magmatic eruptions considered as a precursory phase^4^, it occurred in four main phases beginning with a Plinian eruption (Phase 1), continuing with phreatomagmatic explosions (phases 2 & 3) and ending with pyroclastic flows (phase 4) and caldera collapse^5^. On Santorini island, it led in particular to the destruction of the town of Akrotiri^6^. Tephra fallout^2,7^ and deep-sea homogenites^8^ resulting from the event have been reported across the eastern Mediterranean (Fig. 1). Nevertheless, the exact date of the eruption has been difficult to establish, partly because of the shape of the radiocarbon calibration curve during the eruption period^9–11^. There has also been a long running discrepancy between archaeological dating based on synchronisms with ancient Egypt^12,13^ and certain argued radiocarbon dating ranges^14,15^.

Increasingly accurate knowledge of the Bronze Age eruption suggests that large waves were generated by voluminous pyroclastic flows and mass slumping during the 3rd or 4th phase of the eruption^3^. The most recent modelling has suggested the generation of extreme waves on the north coast of Crete, from several meters up to over 20 m high^16–19^. Nevertheless, while many observations document ash fall from the eruption^2,7,20^ and despite numerous investigations, the evidence for tsunamis remains scarce^21^. The most convincing observations were obtained in eastern Turkey^16^, on the Levant coast^22^ and to the extreme east of Crete^17^ although these deposits still raise many questions^21,23^. Thus, the magnitude and kinematics of the tsunami and its paleogeographic consequences remain largely unknown and the impact of the tsunami is still a subject of debate^23–26^.

**Figure 2:**
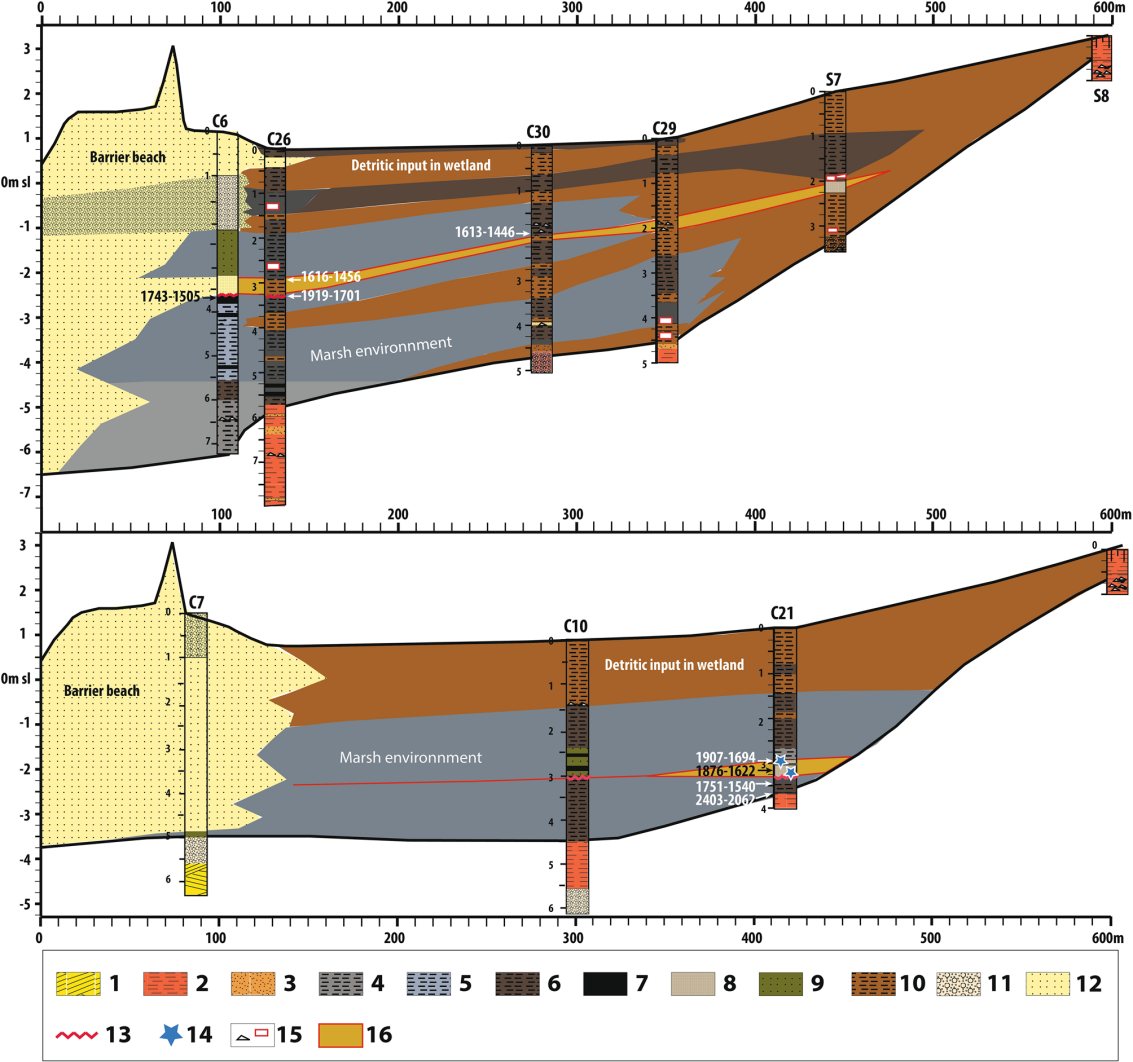
Longitudinal stratigraphy of the Malia marsh and the position of the Late Minoan high energy event, its absolute chronology data (in years BCE). 1. Pleistocene calcarenites; 2. Pleistocene red-ochre sandy silt; 3. Pleistocene gravel and sand; 4. Holocene dark grey organic silty clay (gyttja); 5. Holocene dark grey-blue silty clay; 6. Dark brown silt; 7. Peaty silt; 8. Silty sand layer; 9. Greenish grey sandy clay; 10. Light brown silt; 11. Coarse sand, gravels and stones; 12. Medium grain-size beach and dune sand; 13. Sharp sedimentary contact; 14. Marine microfaunal elements; 15. Limestone fragments (black triangle) and potsherds (red box); 16. Silty or sandy sediments attributable to the time period of the Late Minoan high energy event.

In this paper, we report the results of a systematic geomorphological and sedimentological survey from a small coastal marsh immediately to the west of the Minoan town of Malia (“Supplementary Text” and Figs. S1, Figs. S2) on the Northern coast of Crete. Following a first field campaign which revealed the potential of the marsh deposits for palaeoenvironmental research and a core (C6) that left open the hypothesis of erosion of the marshy deposits by the Minoan tsunami^27^, we proceed with a complementary core drilling survey in the archaeological area to undertake new microfaunal and sedimentological analyses. Shore-landwards transects were drawn to describe the stratigraphic architecture of the Holocene fill (Fig. 2) in order to determine whether the Bronze Age tsunami impacted coastal environments at this location and, if so, how extensive this tsunami might have been.

## Results

### Palaeogeographic reconstruction of the Malia marsh

**Figure 3:**
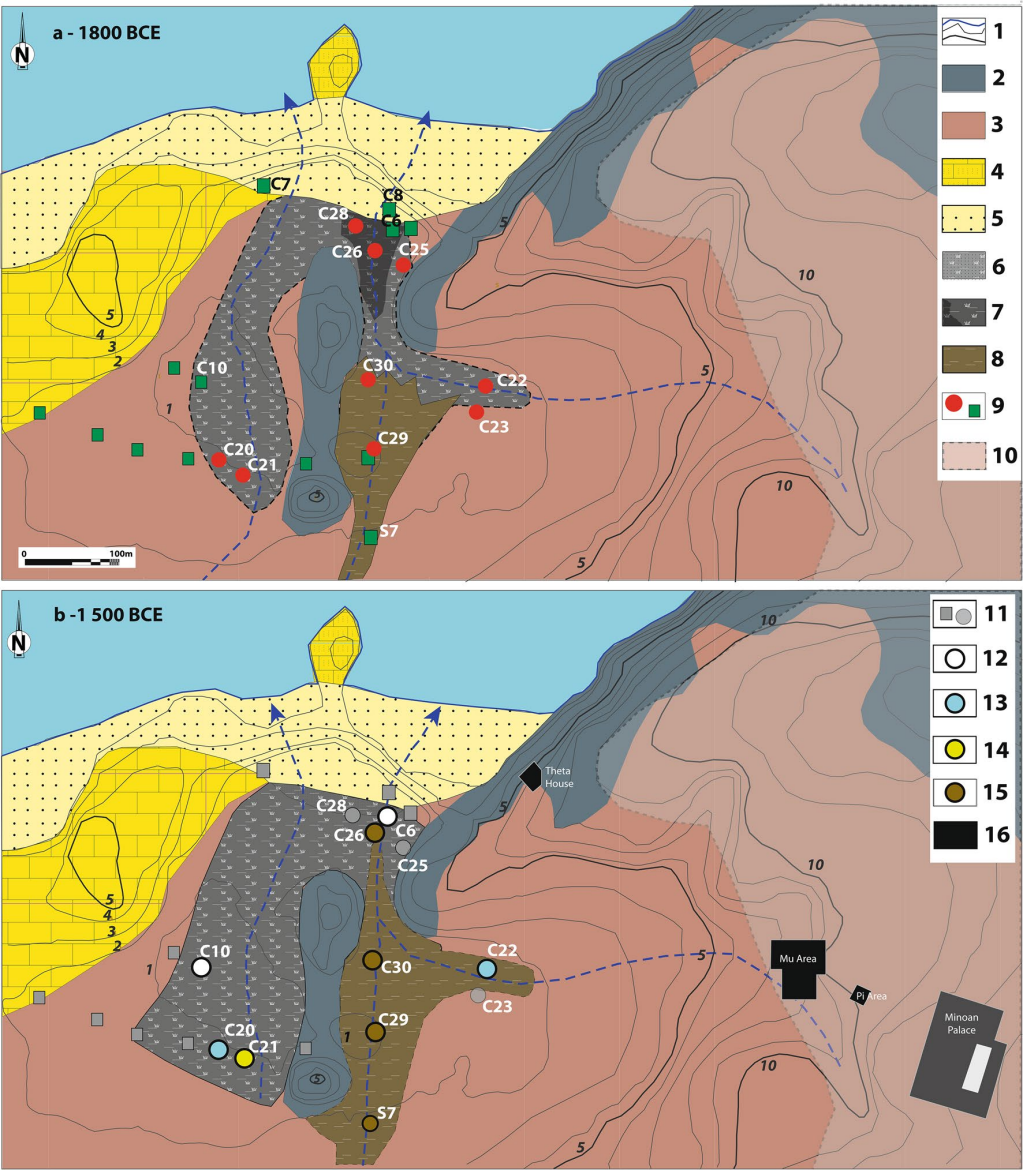
The wetland of Malia before (a: 1800 years BCE) and after (b: 1500 years BCE) the Bronze Age eruption of the Santorini volcano. A- 1. Hypothetical coastal line (blue) and contour line (grey, 1 m eq., elevation indicated for 1–5 m and 10 m contour lines). 2 Cretaceous limestone (Sidheropetra); 3. Pleistocene deposits; 4. Pleistocene Calcarenite; 5. Beach barrier sand; 6. Sandy marshy deposits; 7. Silt to silty-clay organic sedimentation: marshy deposits. The dark grey applet on (**a**) corresponds to the limit of the marsh deposits c. 5000 BCE; 8. Light brown silt: colluvial and fluvial deposits; 9. Core drillings 2015 (red dot) and ante 2015 (green square); 10. Main excavated area of the Minoan town of Malia. (**b**) Evidence of tsunami impact; 11. No clear evidence of tsunami impact; 12. Sharp sedimentary erosional contact; 13. Layer with allochtonous microfaunal marine fossils; 14. Tsunami deposits; 15. Post-tsunami continental sedimentation. 16. Minoan buildings cited in the text including the Minoan palace with its central court (white rectangle). Edited in Adobe Illustrator CS6 2020 version 16.0.

The marsh of Malia is fed by continental aquifers and mainly corresponds to a freshwater reedbed with a more brackish environment immediately behind the barrier beach (3.5 m high). The red-ochre Pleistocene sediments of the Malia plain underwent pedogenesis resulting in a decarbonated brown-reddish soil at the base units of cores C10, C21 and C26 (Fig. 2, symbol 2). At the beginning of the Late Neolithic, c. 5000 years BCE, dark grey organic silty deposits developed to the northeast (Fig. 2). These are interpreted as the sedimentation of an elongated marsh fed by intermittent streams behind a coastal barrier formed during the Holocene marine transgression (Fig. 3a, dark applet 7). From 3000 to 2000 years BCE, an extension of the blue-grey to dark-grey silty organic sedimentation occurs landward along two depressions separated by an elongated hill, south of cores C29 and C21, testifying the development of the wetland (Fig. 3a). These conditions lasted until the beginning of the Late Minoan (LM) period, circa 1630–1525 years BCE. After the eruption (Fig. 3b), the development of dark grey to greenish grey silty organic sedimentation shows a landward progression of the marsh southwest into its current form (Figs. 2 and 3b). From the 1st Millennium BC onwards, the wetland stabilized, with only minor changes.

### C21 core

**Figure 4:**
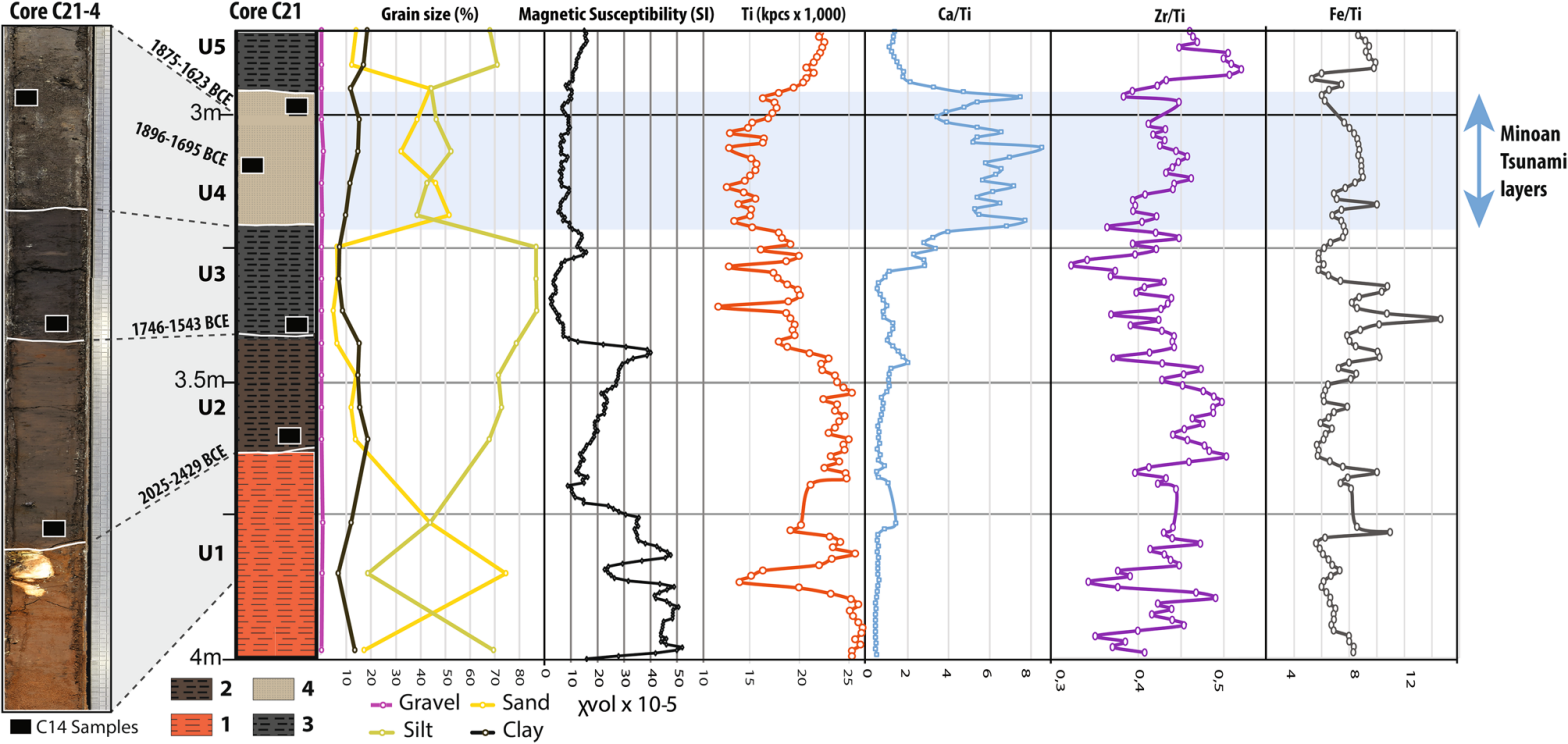
Detail of the C21 core (C21-4 and C21-3 section) with radiocarbon sampling locations (black boxes), grain size, magnetic susceptibility and selection of major elements and geochemical ratios of clastic sources, carbonate content and redox conditions obtained from XRF core scanner analyses. 1. red-ochre silty Pleistocene sand; 2. Brown silty-clay; 3. Dark grey organic silty clay; 4. Silty sand layer with marine bioclasts.

**Figure 5:**
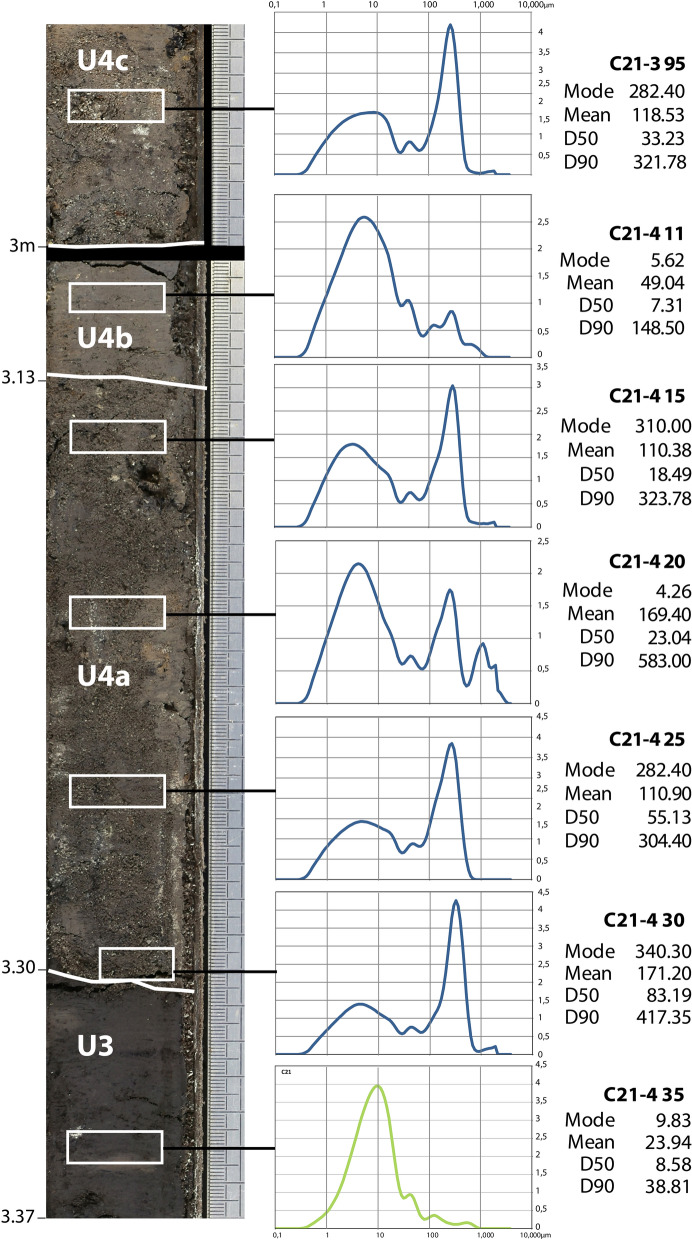
C21 sedimentary facies, grain size distribution and main descriptive statistics of U3 and U4 (high energy deposits). Photographs of C21-4 and base of C21-3 core sections. U3 deposit (in green) are marshy deposits ante-event (vertical depth in m, right-hand scale with elementary unit equal to 1 mm).

In most cores, macroscopic observations of stratigraphy and sedimentary facies failed to show evidence of a high-energy sedimentary event. Core C21, however, to the west of the southern end of the marsh shows a clear signal (Fig. 4). Analysis of the transition from U3 to U5 reveals an abrupt change in the sedimentary facies (Fig. 5). U4 has a sharp basal contact showing an abrupt erosional event with truncation of the underlying dark grey silt marsh deposits (U3). The U4 sand layer is significantly different from these fine silty deposits (D50: 5–10 µm) which both precede and follow it. The main sand unit is 20 cm thick (U4a), characterized by a multimodal composition revealing a mixture of well-sorted medium sand and fine silt (D50: 83–18 µm) with a general normal-graded sequence. It comprises marine mollusks and echinoderms fragments. The next subunit (U4b) corresponds to a silty layer (D50: 7–8 µm) overlain by a final 6 cm layer of medium sand (U4c) with grain size characteristics (D50: 33 µm) very close to the main subunit. The sand deposits of U4 also differ from the surrounding silt deposits due to a decrease in titanium (Ti) and, lower magnetic susceptibility than the preceding marsh deposits, indicating a reduced detrital continental input and pedogenic processes. In contrast, we observe a high calcium (Ca) to Ti ratio indicating a higher carbonate content, even for the intercalated silty layer (U4b) partly related to marine components.

**Figure 6:**
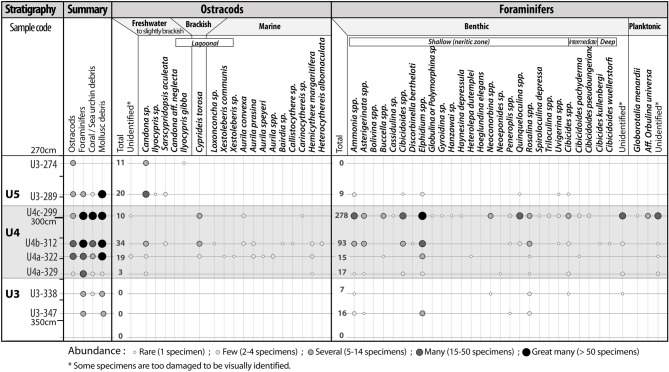
Microfaunal content of samples in C21 Core.

Analysis of microfaunal fossils reveals that the units underlying and overlaying U4 (U3 and U5) contain very few ostracods and foraminifera (Fig. 6; Supplementary Figs. S12–S16). The U4 sand layer however contains an abundance of both. While some tests are broken, sometimes preventing identification, the overall assemblage shows a high species diversity. This is particularly the case for the two upper samples (U4-312 & U3-299). U3-299 contains 278 foraminifera, of which 84.5% are benthic and 8.5% are planktonic. All benthic species are typical of a relatively shallow coastal environment (from lagoon to circalittoral stage) while planktonic species are indicative of more open sea environments. U4-312 is dominated by benthic species indicating a coastal marine environment with almost all the individuals living in shallow waters (infra- and/or circa-littoral stages), howevera few individuals of the genera Cibicides and Cibicidoides are also associated with deeper waters (benthic, intermediate zone). *C. Wuellerstorfi* (1% of the assemblage) and *C. Kullenbergi* (1%) in particular are characteristic of bathyal and abyssal stages.

Ostracod species are much less numerous and diverse than the foraminifera in these sediments. These indicate a lagoonal environment, *Cyprideis torosa* prefers s brackish water and lagoons and constitutes the main species in the three samples, although other lesser indicators of freshwater and other marine environments were also present.

### Origin of the coarse-grained deposits in core C21

Distinction between storm and tsunami deposits remains difficult and a subject of debate^28–30^. Nevertheless, the observations made at Malia offer solid arguments in favour of tsunami deposition. The high energy deposits of C21 mainly show a 20 cm structureless sand layer close to the current beach sediment (Supplementary Fig. S11) comprising coastal biogenic elements (U4a) indicating that the beach was the main sedimentary source. Several other characteristics of tsunami deposits were observed^31,33^. The deposits were found a long distance inland, more than 400 m from the LM coastline (Fig. 2), with a strong basal unconformity, normal grading, bimodal grain size distribution and a high carbonate content. Furthermore, the mixture of intact and broken marine microfossils from both shallow coastal water and deeper marine environments is also characteristics of tsunami rather than storm deposits^32,33^. In summary, three decisive arguments speak in favour of preserved Late Bronze Age tsunamites within the Malia marsh: (1) the absence of other similar sedimentary events within the Late-Middle Holocene sedimentation of the wetland, (2) the inner position and spatial discontinuity of the deposits, and (3) the chronology of the sand units comprising allochthones benthic fauna (see dating section).

The grain-size distribution, ranging from fine to medium sands (Fig. 5), indicates that the flow velocity was not high but sufficient to transport sand grains in suspension. The clear upward fining from U4a to the thin silty layer U4c probably results from the phase when the tsunami energy suddenly decreased, i.e. during a settling phase preceding the backwash or a low-energy backwash. Such sedimentation figures are often described in tsunami sequences^31^, even in small embayments^32^. The thin upper silty layer (U4c) could indicate two successive run-up deposits at C21 separated by a short backwash.

### Other core evidence of tsunami impacts

**Figure 7:**
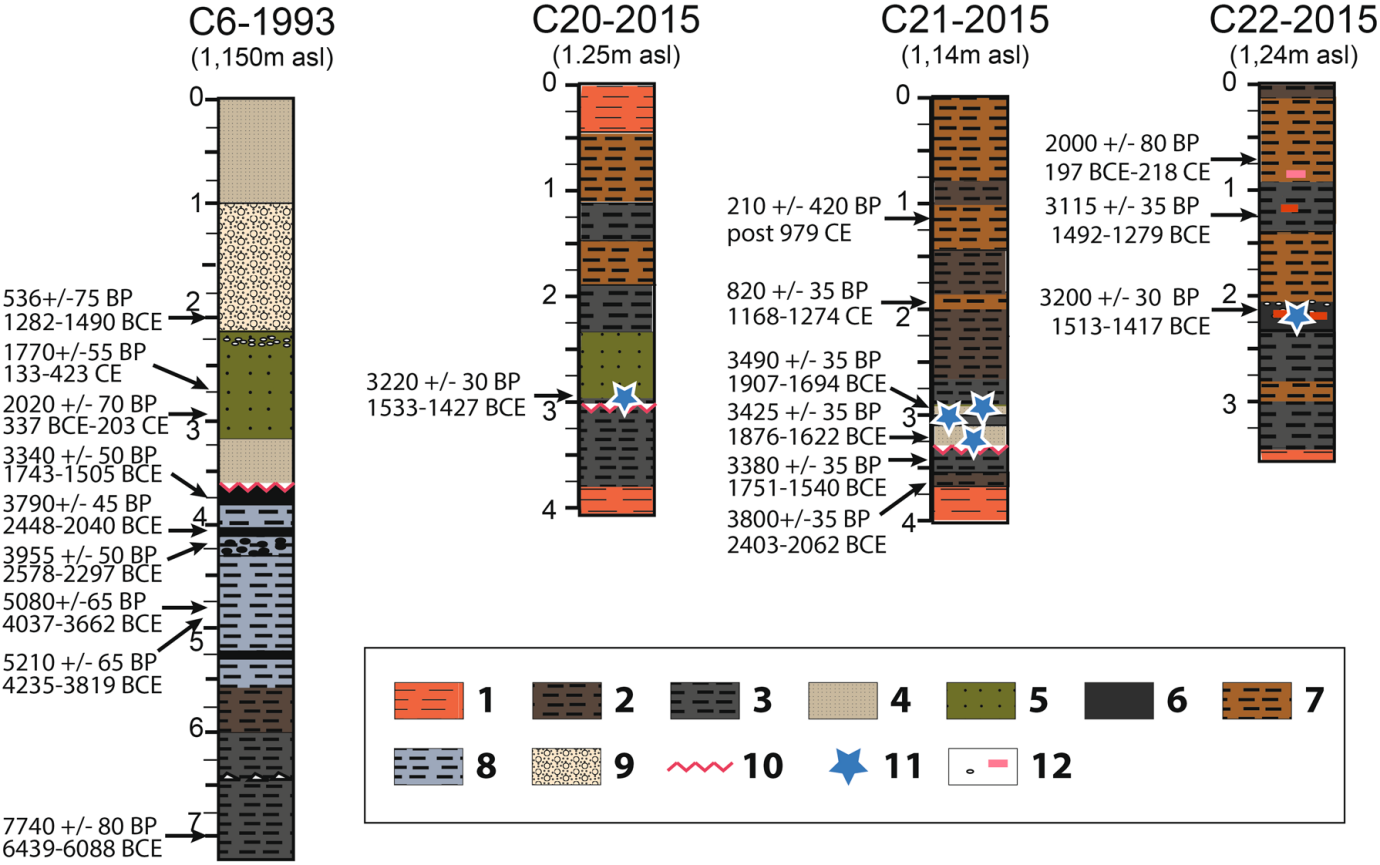
Cores showing specific sedimentary features associated with the Late Bronze Age high energy event. 1. Red-ochre silty sand Pleistocene; 2. Brown silty-clay; 3. Dark grey organic silty clay; 4. Silty sand layer with marine bioclasts; 5. Greenish grey sandy clay; 6. Compact and dense dark clay with many archeological artefacts; 7. Brown silt; 8. Dark grey-blue silty clay; 9. Coarse sand, gravels and stones; 10. Truncation of marshy deposits; 11. Marine microfaunal elements; 12. Limestone fragments (white dot) and potsherds (red line).

At the fore-shore, only C6 shows evidence of the basal non-conformity interpreted as the product of the tsunami in C21^27^. Evidence of tsunami deposits was only found at the south and east end of the marsh (in C20 and, C22 in addition to C21, Fig. 7). In C20, the general stratigraphic organization is close to that in C21 (located 20 m west), but there is no sand layer intercalated in the marshy sedimentation. We observe just a slight change in the color of the sedimentary facies (greenish-grey silt) intercalated within the greyish silt. Taken only two centimeters below a well-dated radiocarbon sample (1605–1425 BCE), one silt sample shows an assemblage very close to that observed in the sandy layer of C21. It comprises 57 foraminifera specimens often broken or very abraded corresponding to benthic (80%) and planktonic (5%) species, while a smaller assemblage of ostracods (14 specimens) is dominated by specimens belonging to a ubiquitous genus (Cladona). A sample taken from the underlying marshy sediment was also characterized by the absence of microfaunal content consistant with the corresponding layer in C21.

Core C22, located at the eastern end of the marsh, at the bottom of the Minoan town provides further evidence of marine microfauna, including 119 specimens of foraminifera belonging to benthic species representing an infra-littoral stage (Supplementary Fig. S12). Between 200 and 227 cm below the surface, it displays a silty sedimentation which shows massive orange–brown silt comprising fine sands with very small gravel, shell fragments, millimeter-sized fragments of ceramics and several charcoal particles. This very compact layer results from a mixing of sediments from different contexts and a reworking of largely anthropogenic sediments. It was radiocarbon dated 1526–1417 BCE.

Observation from the other well-dated cores (C23, C25, C26, C28, C29, C30) shows a lack of clear macroscopic evidence of truncation of the Holocene sediment and even a very thin sandy layer is absent. Moreover, for the two other cores that were analyzed at high-resolution (C26, C30), no significant changes attributable to a high energy event were recorded (“Supplementary Text” and Figs. S4, S5, S9, S10). The sedimentary succession observed in core C26 (3.5–2.5 m) shows very slight changes in color of the sediment (Supplementary Figs. S9, S10). A more yellowish-brown silty sedimentary unit (3.27–2.98 m) is intercalated within the greyish layer and two units dated 1906–1743 BCE and 1616–1458 BCE, respectively. Moreover, the grain-size, magnetic susceptibility and the geochemical analyses undertaken did not show any significant change for this unit. In particular, there is no detectable change in the carbonate content or sedimentation rate, plus a sample, taken from the upper gyttja layer dated 1616–1458 BCE, does not contain any microfaunal remains. In C30, continuous macroscopic sedimentary observations also showed a very tenuous change in the color of the sediment. From 2.2 to 2.0 m deep, a more yellowish–brown silty sedimentary unit is intercalated in the greyish sediment. It corresponds to a sandier deposit, but the geochemical analysis shows no change in relative carbonate content. The upper unit (2.0–1.80 m) is coarser and show peaks in carbonate content (1.95–1.85 m), corresponding to numerous limestone fragments. Again, microfaunal analyses of this unit, dated 1613–1447 BCE, reveals an absence of ostracods and foraminifera.

### Dating the tsunami event

Attempts to directly date tsunami deposits by radiocarbon methods are open to problems associated with the reworking of older organic matter and biogenic artifacts by the run-up and backwash of extreme waves. Dating of the non-mixed deposits above and below the tsunamite is the method that provides the most likely timeframe for their deposition^34^. The marshy lower layer (U3) is dated to 1751–1540 BCE in C21, and to 1743–1505 BCE in C6 (Table 1 and Table S1), providing a consistent dating result for the possible calibrated range of sediments immediately prior to the tsunami. Dated charcoal samples from the sand layer of C21 produce date ranges which could extend a little earlier, but which are still in overlap with the other calibrated ranges, at 1876–1622 and 1920–1694 BCE from bottom to top respectively. This inverted pattern and slightly older dating possibilities are not surprising for what is probably reworked charcoal in the high energy tsunami deposits. It was not possible to obtain a date immediately after the event on C21, probably because of erosion or a local hiatus in sedimentation. But, in the C20 and C22 cores, dating of millimeter-sized charcoal fragments in post-truncation layers gives ages of 1533–1427 and 1513–1417 BCE respectively. These dates come from charcoal in swampy or anthropogenic sediments that were put in place soon after the event. They are the earliest candidates for dating the end of the event. From these dates, we use the function “OXcal combine” to model the pre- and post-event sequences (Supplementary Figs. S6, S7). At 95% confidence ranges, there is no overlapping of the two sequences. For the pre-event layer, we obtain a possible time-window of 1744–1544 BCE (with 87.6% probability in the period 1741–1606 BCE) while for the post-event layer, we obtain the interval 1509–1430 BCE. Additionally, the dates obtained at Malia have some overlap with two dates obtained at Gölhisar Lake in Turkey for the peat immediately underlying the Bronze Age tephra layer, of 1744–1431 BCE and 1611–1415 BCE^35^, providing an Oxcal combined date of 1612–1436 BCE (Supplementary Figs. S8 and Table S2).

**Table 1:** Radiocarbon dates of samples attributed to the Minoan tsunami deposits in their chronological context in Malia (pre- and post-tsunami) and in the wider Eastern Mediterranean.

Site	Core trench	Depth (m)	Material	^14^C age	Error 1σ	Error 2σ	Cal. BCE 1σ	Cal. BCE 2σ	Recalibration BCE	References
Malia (Crete) *Tsunamites*	C21	2.99	Charcoal	3425		35	1865–1641	1876–1622		This study
Malia (Crete) *Tsunamites*	C21	3.22	Charcoal	3490		35	1880–1751	1920–1694		This study
Malia (Crete) *Pre-tsunami*	C6	3.755	Peat	3340		50	1682–1537	1743–1505		This study
Malia (Crete) *Pre-tsunami*	C21	3.44	Gyttja	3380		35	1736–1621	1751–1540		This study
Malia (Crete) *Pre-tsunami*	C21	3.67	Gyttja	3800		35	2290–2149	2429–2065		This study
Malia (Crete) *Post-tsunami*	C20	2.97	Charcoal	3220		30	1507–1148	1533–1427		This study
Malia (Crete) *Post-tsunami*	C22	2.11	Charcoal	3200		30	1499–1444	1513–1417		This study
Didim (Turkey) *Tsunamites*	C1	1.15	Benthic foraminifera	3837	88	NP	1930–1706	NP	2070–1413	Minoura et al. (2000)^16^
Didim (Turkey) *Tsunamites*	C1	1.15	Benthic foraminifera	3886	86	NP	1991–1759	NP	2134–1474	Minoura et al. (2000)^16^
Fetihye (Turkey) *Tsunamites*	C2	1.6	Marine shell	4303	79	NP	2562–2351	NP	2683–2006	Minoura et al. (2000)^16^
Palaikastro (Crete) *Tsunamites*	PR1	NA	Bone (cattle, collagen)	3310		35	NP	NP	1684–1503	Bruins et al. (2008)^17^
Palaikastro (Crete) *Tsunamites*	PR2	NA	Bone (cattle, collagen)	3390		35	NP	NP	1867–1544	Bruins et al. (2008)^17^
Palaikastro (Crete) *Tsunamites*	PR2	NA	Shell (Patelidae)	3790		35	NP	NP	1817–1303	Bruins et al. (2008)^17^
Caesarea (Israel) *Tsunamites*	C1	0.9	Foraminifera	3610		40	NP	1660–1460	1821–1316	Goodman-Tchernov et al. (2009)^22^
Caesarea (Israel) *Tsunamites*	C2	1.3	Foraminifera	3640		40	NP	1680–1490	1866–1396	Goodman-Tchernov et al. (2009)^22^

Calibrations or recalibrations are based on OxCal v4.3.2 and datasets IntCal-20 and Marine20 for foraminifera and marine shells (see “Supplementary Text”).

Comparison of these dates with new radiocarbon dates obtained from archaeological contexts in the town of Malia shows good agreement. Of five radiocarbon dates from LM IA layers at Malia (Table 2), two were obtained from short-lived samples from a mature LM IA phase context in the Pi excavation area^36^ (Fig. 3B) and provide a calibrated age range of 1633–1501 BCE. The archaeological material in this phase is contemporaneous with that of the last period of occupation at Akrotiri on the island of Santorini^6^. Thus, this archaeological phase dating to the mature phase of LM IA is broadly contemporaneous with the Minoan eruption of the Santorini volcano. We can conclude that the most probable time bracket for the high energy event identified in the Malia marsh is during the sixteenth century BCE. This is consistent with date ranges based on calibrations of key radiocarbon dating evidence from immediate pre-eruption contexts on Santorini to IntCal20 and also with radiocarbon dates from the site of Tell el-Ajjul, Gaza, found in association with Santorini pumice, and compatible with archaeological interconnections with Egypt^12^. Moreover, recent high resolution studies of Antarctic and Greenland ice have begun to exclude the possibility of a seventeenth century BCE date for the Thera eruption and suggest instead a focus on the period 1570–1500 BCE to look for volcanic ash in the Greenland ice cores^37^. This move towards the sixteenth century BCE is in line with our dating results for the Malia tsunami. Unfortunately, because of the plateau in the calibration curve and the standard deviation, these results emphasize that palaeoenvironmental records with higher resolution will be needed to indisputably pinpoint the exact date of this event^9^.

**Table 2:** Available radiocarbon dates of samples from the archaeological layers attributed to the LM-1A phase in Malia.

Excavation area	Archaeological context	Archaeological chronology	Material	^14^C age	Error 2σ	Cal 2σ	Sample code	References
Epsilon	Square A4, 4th level, post-destruction	Late Minoan 1A	Charcoal	3200	250	2132–836 BCE	Gif-256	Delibrias et al. (1970) ; Pelon et al. (1992)
Abords Nord-Est	Room 20.2	Late Minoan 1A (early or mature)	Charcoal *Amygdalus Communis*	3315	35	1886–1504 BCE	Lyon-5070 (SacA-11021)	Darcque (2014)^39^
Abords Nord-Est	Destruction of building 10B (level 11)	Late Minoan 1A (late phase) or Late Minoan 1B (early phase)	Charcoal *Amygdalus Communis*	1770	30	1505–1397 BCE	Lyon-5072 (SacA-11023)	Darcque (2014)^39^
Pi	Statigraphical unit 4.030, space 11	Late Minoan 1A (mature)	*Olea europaea*	3290	30	1622–1501 BCE	Lyon-17504 (SacA-60294)	This study
Pi	Stratigraphical unit 4.063, space 16	Late Minoan 1A (mature)	*Olea europaea*	3290	30	1622–1501 BCE	Lyon-17505 (SacA-60295)	This study

### Tsunami impact at Malia

Run-up height, inundation distance and spatial extension of the erosion and deposition pattern are the key elements to determine the impact of a tsunami. The observations made in Malia suggest that the tsunami was mainly characterized by lower scale erosion of the marshy sediment and deposition to the south and east end of the marsh. This suggests a predictable decrease in the wave energy towards the south and east of the marsh, perhaps in connection with a northwest-southeast oriented wave train. The decreasing force seen at C22 could be explained by localized effect of a slope of about 10 m separating the marsh from the plateau of Malia town, contrary to the area of C21 where the slopes of the Pleistocene fan gradually rise northwards. We note that overall, the event layer is thin and has not been observed (to date) further inland. After the eruption, light brown silt deposits (C30, C29, C22, C26) indicate an increase of detrital continental input to the east (Fig. 3B), whereas to the west the persistence of organic silt and peat deposits indicate the relative weakness of colluvial and alluvial inputs.

The archaeological excavation closest to the barrier beach (with LM1A occupation) is the Theta House, around 6 m asl^38^. The Neopalatial levels were eroded and badly preserved but some Neopalatian vases found during the excavation were found complete. Nothing in the record of the Neopalatial material suggests a chaotic layer of destruction that might indicate an effect from the tsunami. Unfortunately, this excavation was carried out some time ago and is too inaccurate to be conclusive one way or the other about the impact of the tsunami. However, so far we must underline that any evidence of the tangible impact of a tsunami is also missing from numerous excavations conducted in the center of the Malia town. The observed destruction of the late LM-1A or early LM-1B is unrelated to the impacts of the tsunami as the destruction of building 10B at the north-eastern edge of the palace falls within the 1505–1397 BCE range (Table 2), later than the eruption of Santorini^39^. Therefore, the tsunami did not impact the main part of the Minoan town established on the plateau, or most of the Minoan settlements of Northern Crete^40^ including the centre of the town of Palaikastro^41^. As the center of the Minoan town is located about 400 m to the east and 8–14 m above the current sea level (asl), it suggests a maximum height for the run-up surge of less than 8 m asl. The definition of the LM relative sea level is difficult in an active tectonic context and the position of the LBA shoreline remains highly hypothetical. Nevertheless, we know that the top of the marsh sedimentation today is around 0.7–0.8 m meters above sea level and it is in equilibrium with the groundwater level which is controlled by the current sea level. This indicates that the marshy deposits are probably ± 1 m in relation to the sea level. As, the marsh deposits preceding the tsunami were around 2.5–1.75 m bsl, this provides an approximation of the Late Minoan sea level (msl). Moreover, this estimation is close to the observations made for eastern-central Crete which suggest a sea level 2–3 m below the present one at the time of the Santorini eruption^42^. Therefore, the maximum run-up of the tsunami was probably less than 10 m above the Late Minoan sea level (msl), and the wave height at the coastline would have been much less. In the flat area of the Malia marsh, the inundation distance was probably up to 500 m, but also with only modest geomorphological consequences.

### Other Bronze Age tsunami candidates and their chronology

Most of the older, less precise reports regarding tsunami deposits have been largely rejected today, such as those from northern Crete at sites in Amnisos^24^ and Gouves^16^ or on the Levant coast^43^. These relied on limited observations making the interpretation of the deposit doubtful^21,23^. Suspected thin tsunami deposits have also been identified in coastal plains of eastern Sicilia^44^ and in north-west Crete^45^, but another origin has been suggested for the former^19^ and the latter is not directly dated. Both also remain difficult to distinguish from storm deposits in sequences comprising several other high energy marine deposits.

Five deposits attributed to the Minoan tsunami have been adequately described^16,17,23,46^ but published dates have been obtained mainly from the tsunami deposits themselves and illustrate the hazards of dating of mixed age samples in tsunami deposits. The results are questionable and wide-ranging (Table 1). On the Turkish coast plain near Didim and Fethiye, marine sand deposits of 10–15 cm thickness, directly overlain by a tephra layer, 1–1.5 m bsl and 60 and 120 m inland of the current coastline respectively^16^. In the case of Fethiye, the dating control is weak because of the very old date associated with the tsunami deposits (2683–2006 BCE). In fact, the attribution to the LBA period is mainly based on the refractive-index measurements and the XRF chemistry of the overlying tephra which is argued to be consistent with the Santorini eruption. The Didim deposit is more convincing. The stratigraphic position is close to the one observed at Fethiye and the dates obtained in the tsunamites are very close to those obtained in the Malia tsunamites, even if the margins of error remain too large.

In the outskirts of the Minoan site of Palaikastro, at the east end of Crete (150 km south of Santorini), an unsorted chaotic layer of coastal gravel, sand, and marine bioclasts, with building stones, ceramic sherds and ash inclusions was interpreted as a tsunami deposit^17^. This deposit extends over 100 m at the top of a low coastal cliff above the Chiona beach, but no evidence further inland has yet been reported. The maximum run-up height was estimated around 9 m asl. However, we cannot exclude that the observed chaotic layer is partly the result of heavy rainstorms^17^ and associated continental flash floods which are frequent in Crete, for these can generate this kind of chaotic layer^23^. The abundant LM IA artefacts (pottery sherds, architectural and faunal remains) show that the wave must have passed through densely inhabited spaces that are now eroded or submerged, but so far there has been no research to attest such a dynamic. Two of the three radiocarbon dates were obtained from archaeological material (animal bones of different ages) from a layer comprising numerous LM IA artifacts. In our opinion these should be considered for their capacity to date the rich local archaeological context rather than the extreme marine event. Moreover, the same layers comprise older marine shells (2550–2000 BCE) and marine gastropods dated to 2000–1300 BCE that are interpreted as “storms or later tsunami [that] could have dropped these shells”^17^ (p. 207), which raises doubts about the unique origin of all the deposits. The older shell assemblages overlap in time range with the older dates associated with the Fethiye tsunami deposits. While in both cases it is argued that these significantly older dates relate to older material mixed up in the tsunami, it is also possible that the assemblage dates to an earlier event.

Tsunami deposits from submerged sediment cores taken offshore from Caesarea, Israel have been suggested to mark the maximum extent of the Minoan tsunami in the East Mediterranean^23^ although these deposits could also have their origin in a landslide-induced tsunami, occurring in the eastern area of the Nile Delta^19^. Radiocarbon date ranges for the up to 40 cm thick submarine deposit at Caesarea are large because of dating uncertainties in the marine reservoir age, but could match the approximate time span for the Minoan eruption and overlap the dates from Malia. Arguably the most secure Minoan eruption tsunami evidence comes from the eastern coast of Santorini itself, where a thick tephra layer from the last eruption phase has been interpreted as reworked by the volcanogenic tsunami, with a reported run up of 6 m asl^46^, quite consistant with the more modest impacts seen at Malia.

To conclude, models proposing wave heights much higher than 8 m asl for northern Crete would seem at this current time to be overestimations and those concluding higher tsunami wave height in northern Crete than in eastern Crete must be questioned^16,18,19^. More precise and localized analysis of run-up heights and inundation extents will allow us to understand the full consequences of the tsunami on cultivated and inhabited areas, but indications from Malia are that the impacts of the Minoan tsunami were relatively modest. The Malia evidence opens the field for new research on the event, specifically by illustrating that tsunami evidence can be found in positions further away from the present coastline than may previously have been considered in numerous coastal plains of northern-central and eastern Crete. These should be the focus for future investigations.

## Materials and methods

In 2015, permission was obtained to proceed with a new core drilling survey in the archaeologically significant area of the Malia marsh, to build on preliminary findings, especially in the eastern part of the marsh, closest to the Minoan town, a full geomorphological study. Eleven 50-mm-diameter cores were obtained from up to a maximum depth of 8 m below the surface using a hand-driven vibrocorer (Cobra TT) and hydraulic extractor. Each core was located using a Leica differential Global Navigation Satellite System (GS15 type). The acquisition of points was carried out in real time kinematic (RTK) using a network (SmartNet) of fixed antennas from the access provider. The points were recorded in the Greek EGSA87 system. The topography of the study area was obtained from a combination of the 1/5000 Greek army plan (GYS) (ref. 9622/2) and archaeological maps from the French School of Athens derived from the digitization of photogrammetric plans made in 1990 by the Polytechnic School of Athens. The reference zero is that of the tide gauge of Piraeus and corresponds to mean sea level 0. The tide range in Malia ranges from 10 to 30 cm. Cores C26 and C25 were duplicated and three cores were extracted with an open open-barrel and described and sampled in the field (C20, C22, C29). For other cores, PVC core liners were inserted inside a rigid one-meter-long core barrel that included a core-catcher. Liners were shipped to the laboratory and split using a vibratory cutter to describe the sedimentary facies’ succession for each core and to sample the selected ones.

50 AMS radiocarbon dates were obtained from gyttja (organic clay) and millimeter-sized charcoal (1–2 mm) material, with 12 AMS dates specifically for the period 2500–1500 BCE. Calibration or recalibration of each date was based on OxCal v4.3.2^47^ and datasets IntCal 20^48^ and Marine20^49^ for foraminifera and marine shells (“Supplementary Text” and Tables S1, S2). We used the same protocol to re-calibrate the dates obtained from just below the Late Bronze age Santorini ash layer from cores obtained at Gölisar Lake in South Turkey^35^ and the available dates of the LM Phase 1A obtained in Malia (Table 2). The chronostratigraphy of the Holocene fill was reconstructed from the description of the successive sedimentary facies and the chronology obtained from radiocarbon dates for each core (Figs. S4–S6).

Three cores were sampled every 5 cm for sedimentological analyses. The grain size distribution was measured by laser diffraction with a Beckman-Coulter LS230 on C21, C26 and C30 cores (“Supplementary Text”). Magnetic susceptibility was used to identify traces of paleopedogenesis and organic matter accumulation, oxidation–reduction and detrital phases enriched in magnetic minerals or in a fine fraction inherited from old fersiallitic soils surrounding the marsh. Each core was measured twice every 0.5 cm using a MS2F Bartington Susceptibility Meter. The value of each measurement was multiplied by 0.5 to correct for variations in sensitivity around the probe head. Resulting values corresponded to the mean of the two corrected measurements and are equal to χvol × 10^–5^ S.

To estimate relative concentrations of major and trace elements in three cores (“Supplementary Text”—Figs. S9, S10), we measured elemental intensity using scanning X-ray fluorescence spectrometry (XRF) via an Avaatech core scanner. XRF measurements do not provided concentrations of elements, but the uncalibrated values produced can be used as estimates of the relative concentrations to provide paleoenvironmental information^50^. Ti is considered as a proxy of terrigenous silicate input as it is strictly of terrigenous origin. It is mainly present in clay minerals while Zr ranks among heavy minerals and is more often associated with coarser silt and sand-size fractions^51,52^ related to high-energy environments^53^ and coastal sandy deposits^54^. We used the Zr/Ti ratio as a grain size proxy to identify less visible changes in the sedimentation process and potential coastal input. Ca indicates detrital carbonate deposition and/or the carbonate precipitation signal. We used the Ca/Ti ratio to normalize changes in Ca relative to a clastic source and to detect authigenic carbonate or marine input because the beach is characterized by medium carbonate sand. Changes in Fe can indicate change in redox conditions in the marsh as precipitation of the Fe oxide signal can relate to the oxygenation processes^55,56^ but it can also be indicative of clastic input. Na, Cl and S were too close to detection limits to be considered.

Microfossil analyses were conducted on 22 samples distributed across five cores (C20, C21, C22, C26, C30) in order to determine microfossil content of the sedimentary layers attributed to the eruption period and to identify allochthonous deposits within the marsh (Fig. S12). For each sample, 7 cm^3^ of sediment was taken from a 5 cm diameter half core and sieved at 50 μm. The sieve residue was dried naturally. Individual fossils were isolated and photographed using a Leica M205C binocular loupe and a conventional scanning electron microscope Zeiss EVO 10 (Figs. S13–S16). Due to the relatively small amount of sediment treated, individuals were systematically counted. The fossil ostracods and foraminifa were isolated and photographed. Their identification and the definition of their ecological characteristics was determined using regional references^57–66^. Taxonomy was defined using the international taxonomic registers WoRMS (World Register of Marine Species) and PESI (Pan-European Species-directories Infrastructure).

## Supplementary Information

Supplementary Information.
