# Supplementary material for: Discovery of a tsunami deposit from the Bronze Age Santorini eruption at Malia (Crete): impact, chronology, extension
Source: Sci Rep. 2021 Jul 29;11:15487. doi: 10.1038/s41598-021-94859-1 (PMC8322394; doi:10.1038/s41598-021-94859-1)
Supplement: Supplementary file 1 — Supplementary Information. [file 41598_2021_94859_MOESM1_ESM.pdf]

## Supplementary Information Text

### Previous research, sediment coring and topography

A first field campaign revealed the potential of the marsh deposits for palaeoenvironmental research (Supplementary Figures S1, S2) and confirmed the absence of an inner harbor at the bottom of the Minoan town[31], contrary to what had been assumed (Supplementary Reference S1). On this initial exploration, core C6 was the only one that left open the hypothesis of erosion of the marshy deposits by the Minoan tsunami. Unfortunately, the sand deposits above the erosional contact were lost and could not be analyzed but the erosion of the marshy deposits was clear. We obtained permission in 2015 to proceed with a new core drilling survey in the archaeological area to complement the previous one.

### Methods

### AMS Radiocarbon dating

The radiocarbon dates were performed in three laboratories (Lyon, Poznan and Beta-Analytic). Of the 50 dates obtained, 5 were rejected due to their inverse stratigraphic position and their inconsistency with the age-depth models (in italic, Tab. S1). They were mainly provided by millimeter-sized charcoals that have migrated in soil profiles as a result of post-depositional processes (2 dates) and two very small fragments of plants too young that probably correspond to reed rootlets (2 dates). Additionally, for the radiocarbon dates obtained from marine samples provided by other studies, we estimate the age reservoir for Mediterranean Sea and the ΔR from the Marine Reservoir Correction Database of Calib. 8.20. For the Aegean Sea we use a ΔR of -48±78 resulting from the average of the two sites available (Supplementary Reference S2) and for the Caesarea, we use -139±73 resulting from the average of the six nearest data available (< 40km, Supplementary Reference S3). Age-depth modelling was performed using Bacon 2.5.1. (Supplementary Figures S3 to S5) (Supplementary Reference S4). Combined dates were obtained using Oxcal R-combine function[49] (Supplementary Figures S6 to S8)

### Sedimentological and geochemical analyses

### Grain-Size Measurements

Organic matter was removed from the samples using hydrogen peroxide (35%). Sediment samples were dispersed with hexametaphosphate sodium (5‰). The grain size distribution (ranging from 0.04 to 2000µm) was measured by laser diffraction with a Beckman-Coulter LS230. Grain size of coarse material (>2mm) was determined using sieves ranging from 2 to 12mm.

### Scanning X-Ray fluorescence spectrometry (XRF)

Core-scanner XRF measurements have been performed in two different laboratories. Measurements were done every 5mm for C30 and C26 (Fig. S10, S11) at EDYTEM (CNRS-University of Savoie Mont Blanc) and every 10mm for C21 at EPOC (CNRS-University of Bordeaux). In both cases, an X-Ray beam was generated with a rhodium anode and a 125µm beryllium window, which allows a voltage range from 7 to 50 kV and a current range of 0 to 2 mA. The analytical settings were adjusted at 10 kV and 1 mA to detect light elements and trace elements (*i.e.* Sr, Rb, Zr, Br, Pb, Cu) were detected in a second run, performed at 30 kV for 0.75 mA. Each measurement is expressed in counts per second (cps).

**Results**

**Fig. S1. Geographical context of the palaeoenvironmental investigations at Malia.** Photo of the Malia marsh and the Minoan town of Malia (©L. Lespez).

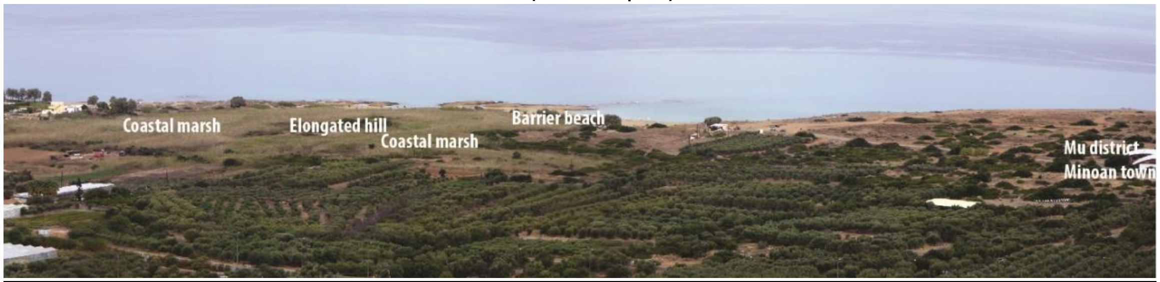

**Fig. S2. Geomorphological map and location of the core drillings obtained in the Malia marsh.** 1. Current coastal line (blue) and contour line (grey, 1m eq., main contour line (eq. 5m) bold grey); 2. Streams and ditch: intermittent (dashed line) and perennial flow (solid line); 3. Cretaceous limestone (Sidheropetra); 4. Pleistocene silty deposits; 5. Pleistocene calcarenite; 6. Beach barrier sand; 7. Sandy marshy deposits; 8. Marshy deposits; 9. Colluvial and fluvial deposits; 10. Core drillings 2015 (red dot), ante 2015 (green dot). Edited in Adobe Illustrator CS6 version 16.0.

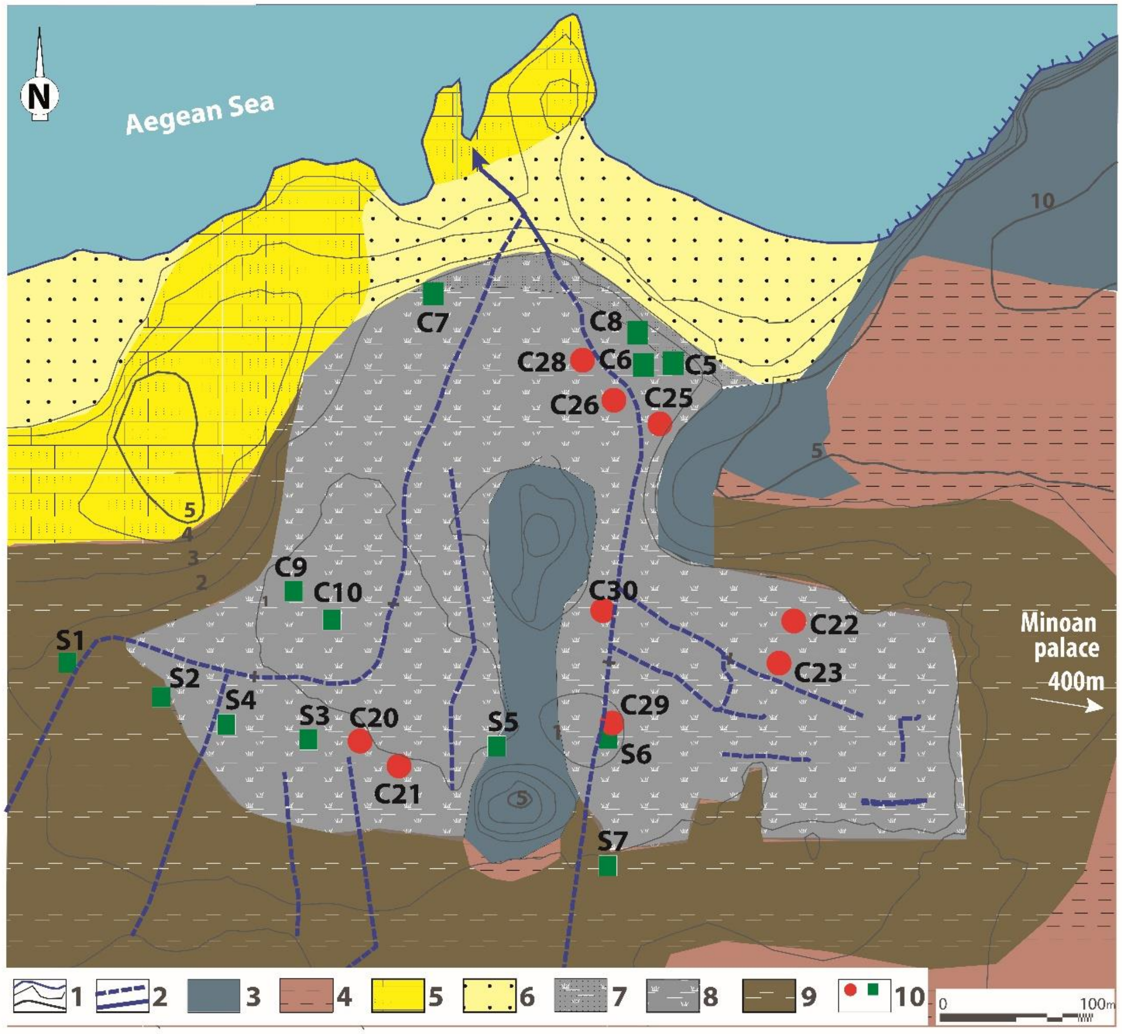

**Fig. S3. Age-depth model of C21 core.** Age-depth modelling was performed using Bacon 2.5.1 and radiocarbon dates from Table S1.

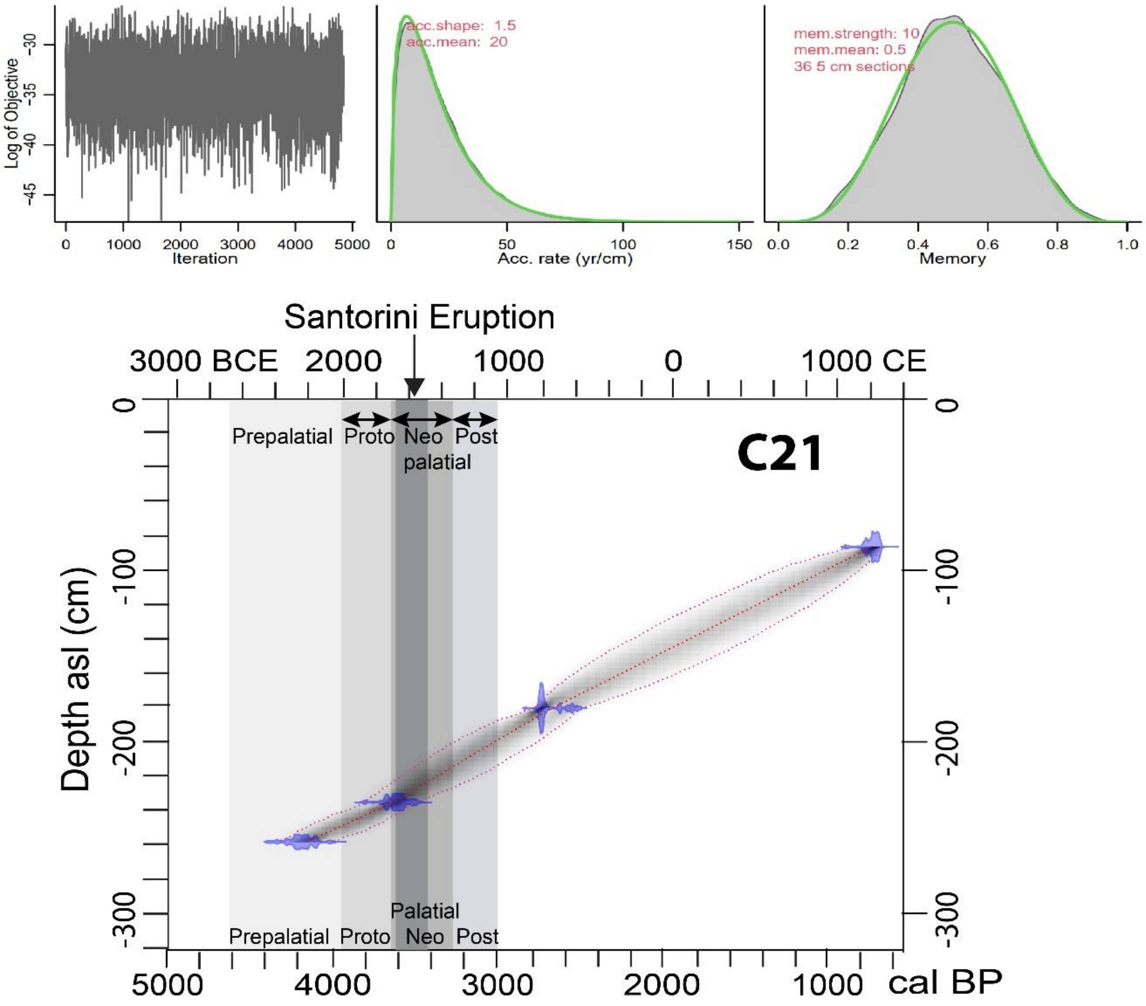

**Fig. S4.** Age-depth model of C6 core. Age-depth modelling was performed using Bacon 2.5.1 and radiocarbon dates from Table S1.

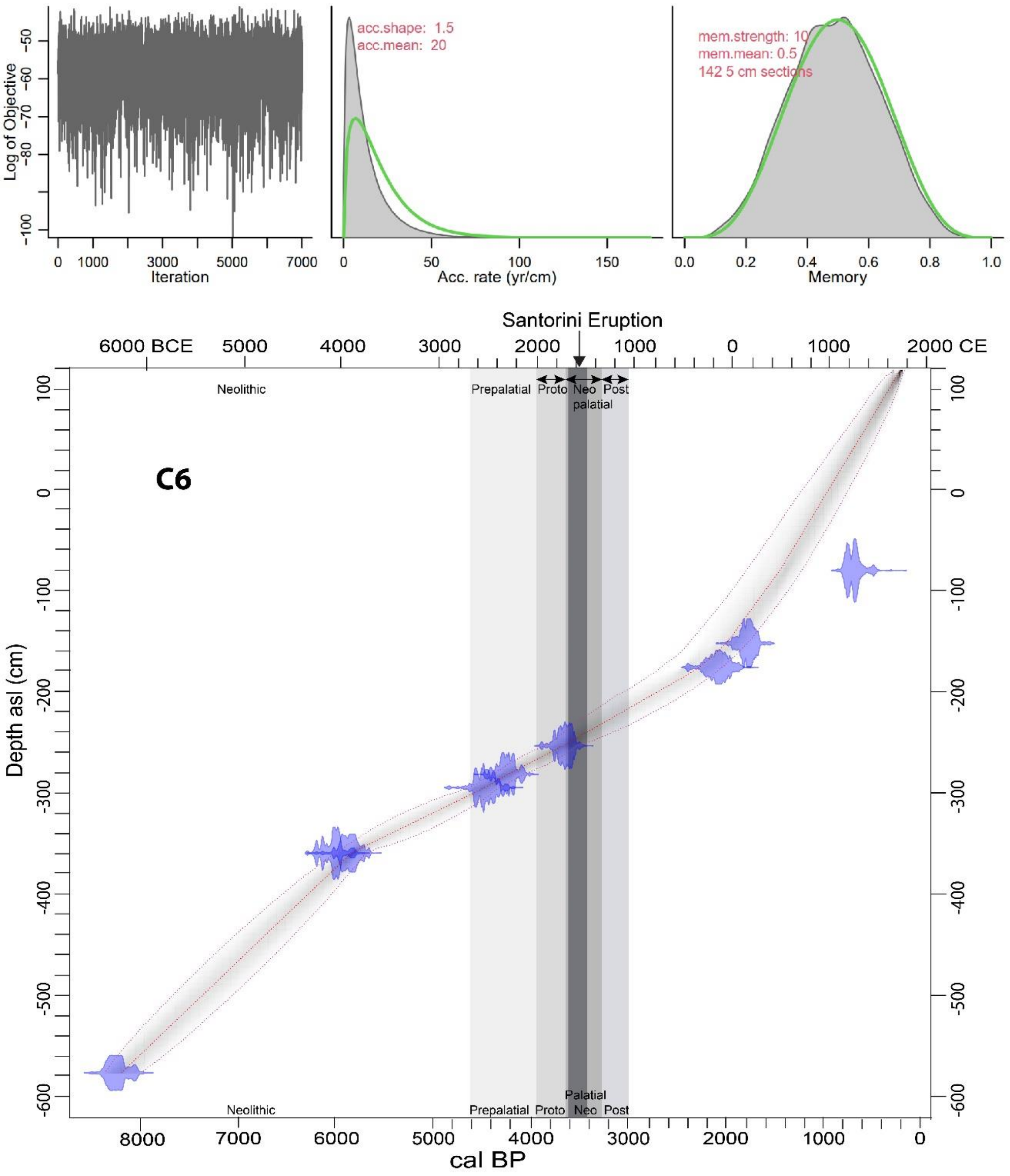

**Fig. S5. Age-depth model of C26 core.** Age-depth modelling was performed using Bacon 2.5.1 and radiocarbon dates from Table S1.

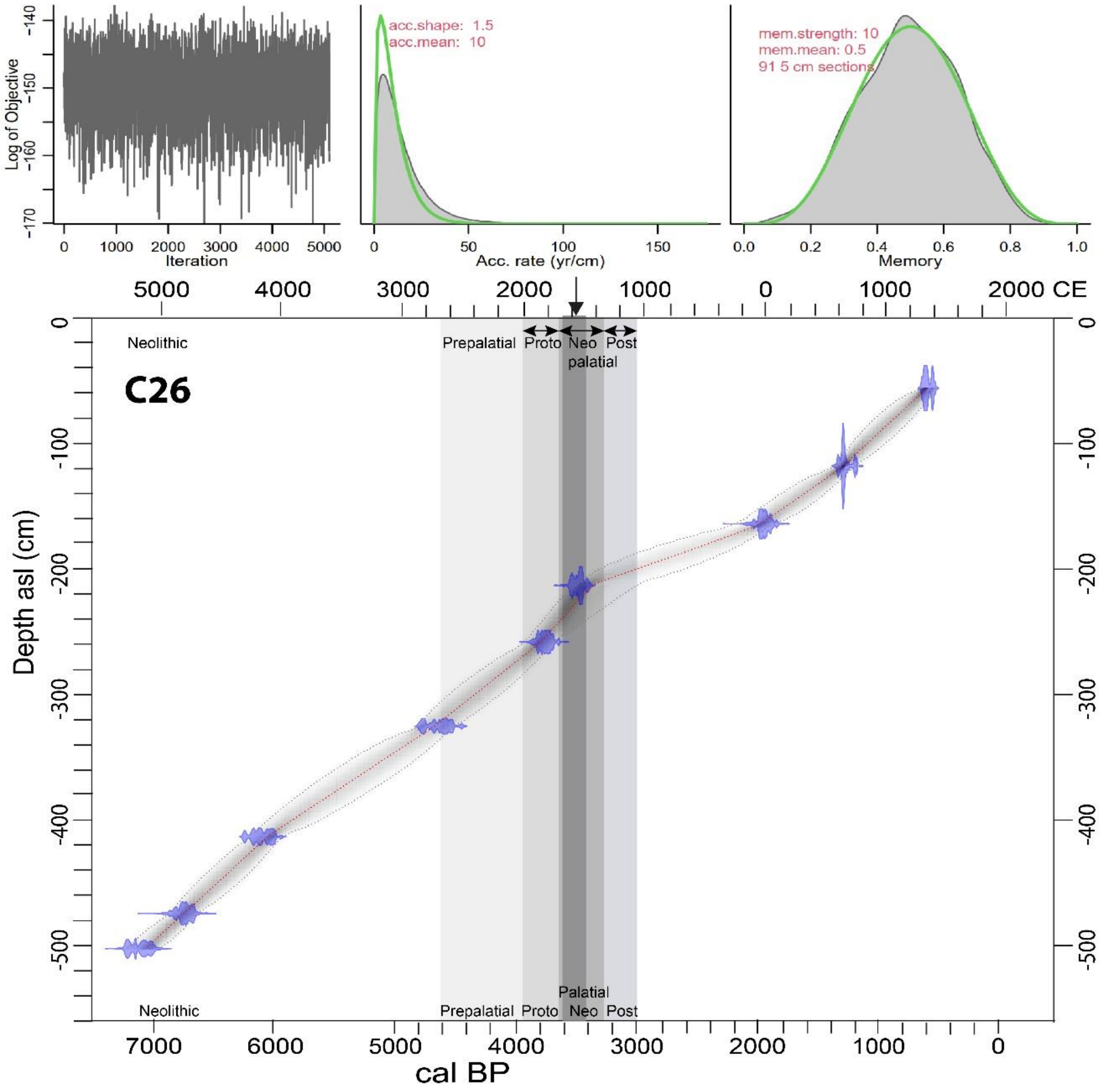

**Fig. S6. Pre-tsunami combines dates.** From C6 (3.755m), 3340 +/- 50 (Lyon-7116), and C21 (3.44m) 3380 +/-35 (Poz 95778).

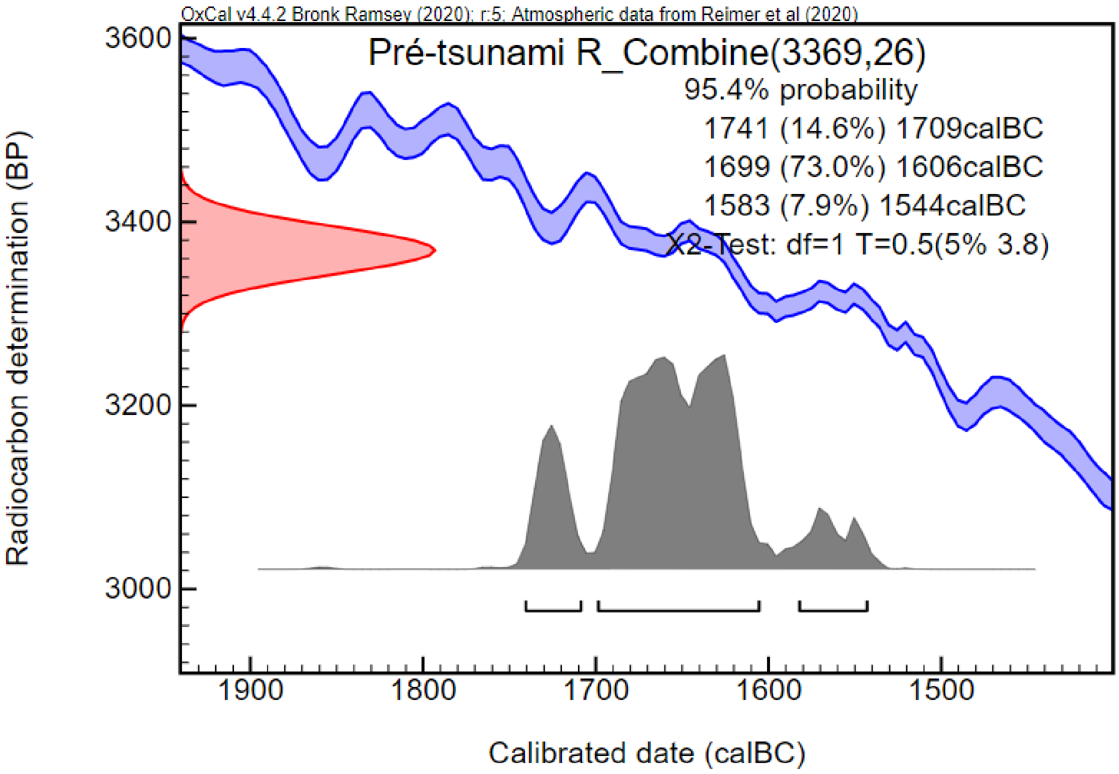

**Fig. S7. Post-tsunami combines dates.** From C20 (2.67m), 3220 +/- 30 (Poz-85442), and C22 (2.11m) 3200 +/-30 (Poz 96175).

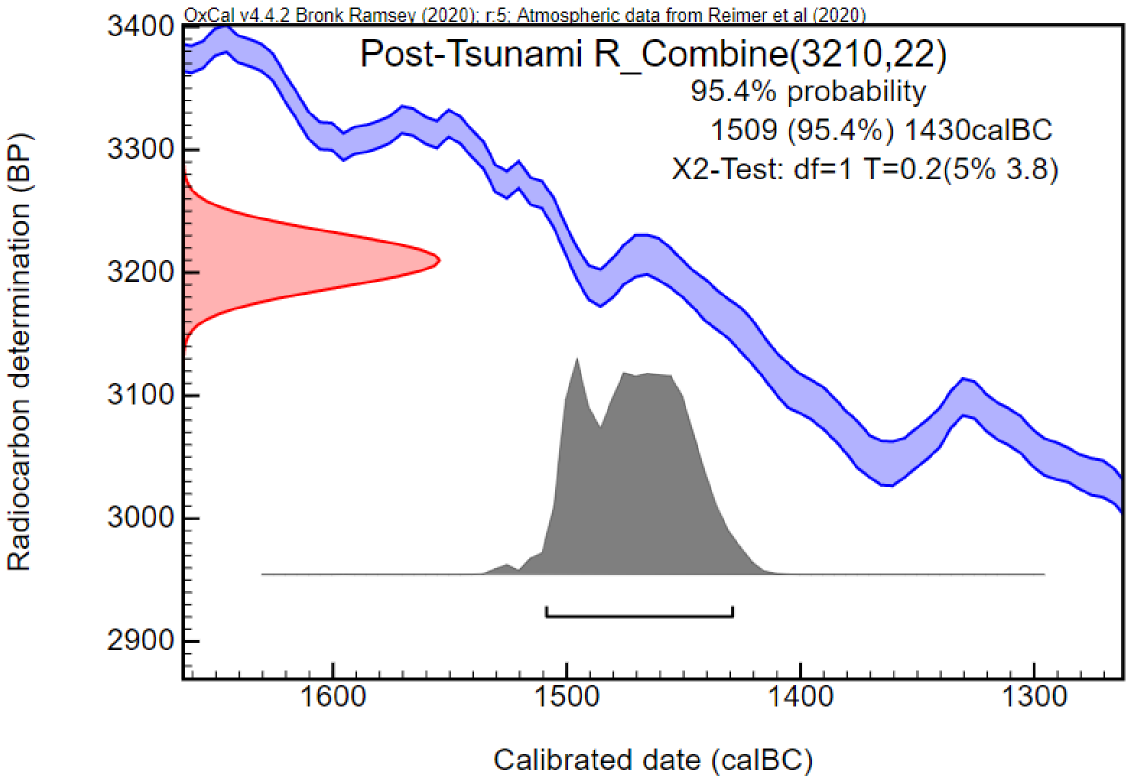

**Fig. S8. R-combine Oxcal Gölhisar underlying tephra layers** (dates in Eastwood et al., 1999)

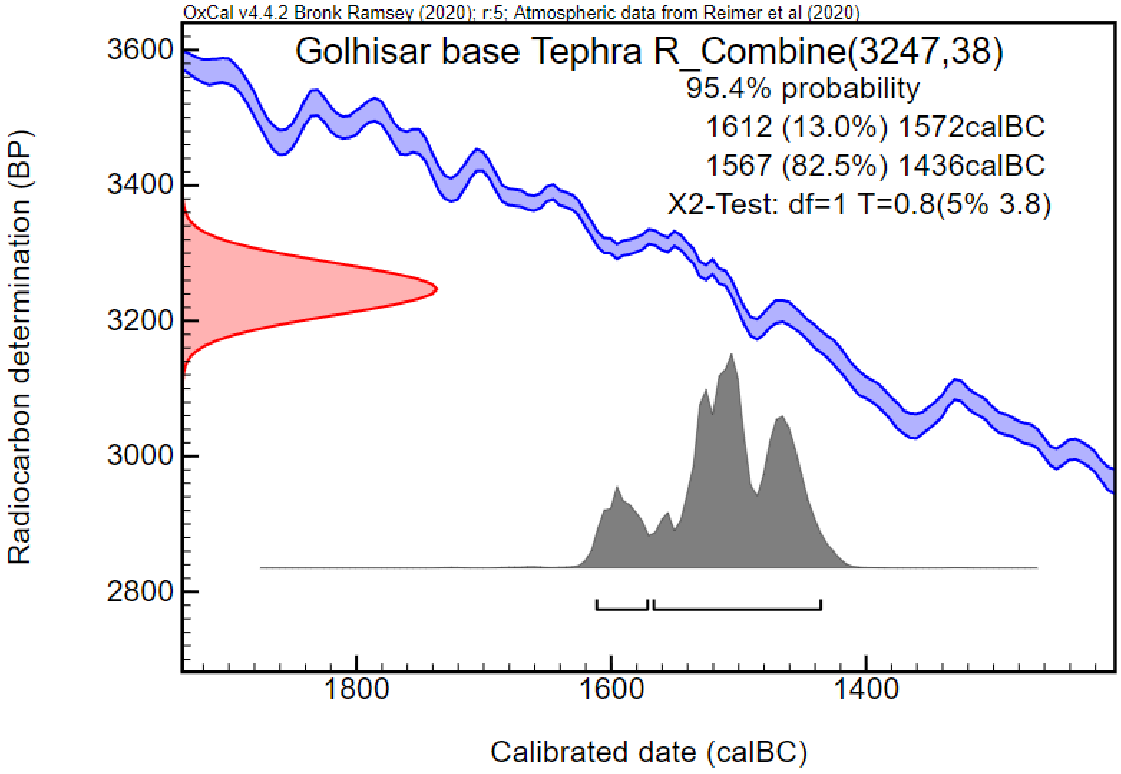

**Fig. S9. C26 core selected analyses with radiocarbon sampling locations (black boxes), grain size, magnetic susceptibility and selection of major elements and geochemical ratios of clastic sources, carbonate content and redox conditions obtained from XRF core scanner analyses.** 1. Sand. 2. Red-ochre Pleistocene silt; 3. Dark grey organic silt; 4. Brown silt.

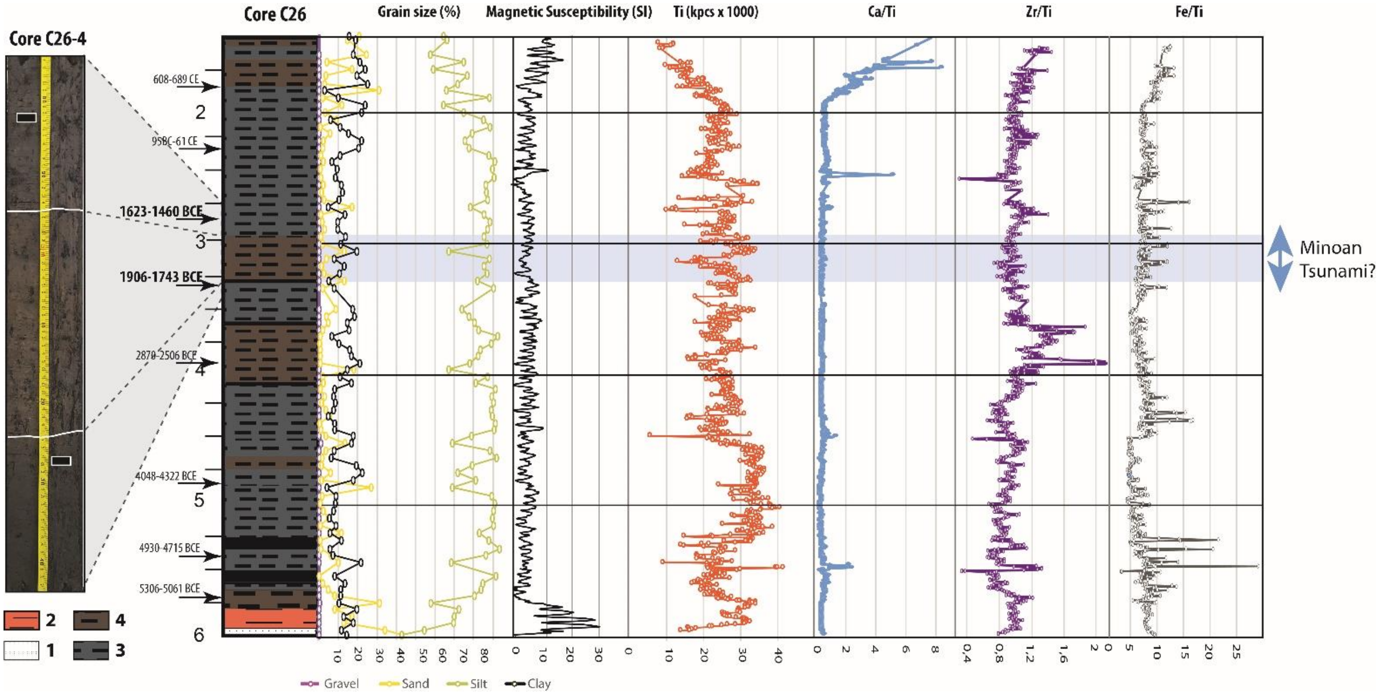

**Fig. S10. C30 core selected analyses with selected analyses with radiocarbon sampling locations (black boxes), grain size, magnetic susceptibility and selection of major elements and geochemical ratios of clastic sources, carbonate content and redox conditions obtained from XRF core scanner analyses.** 1. Dark grey organic silt. 2. Brown silt; 3. Light brown silt; 4. Limestone fragments.

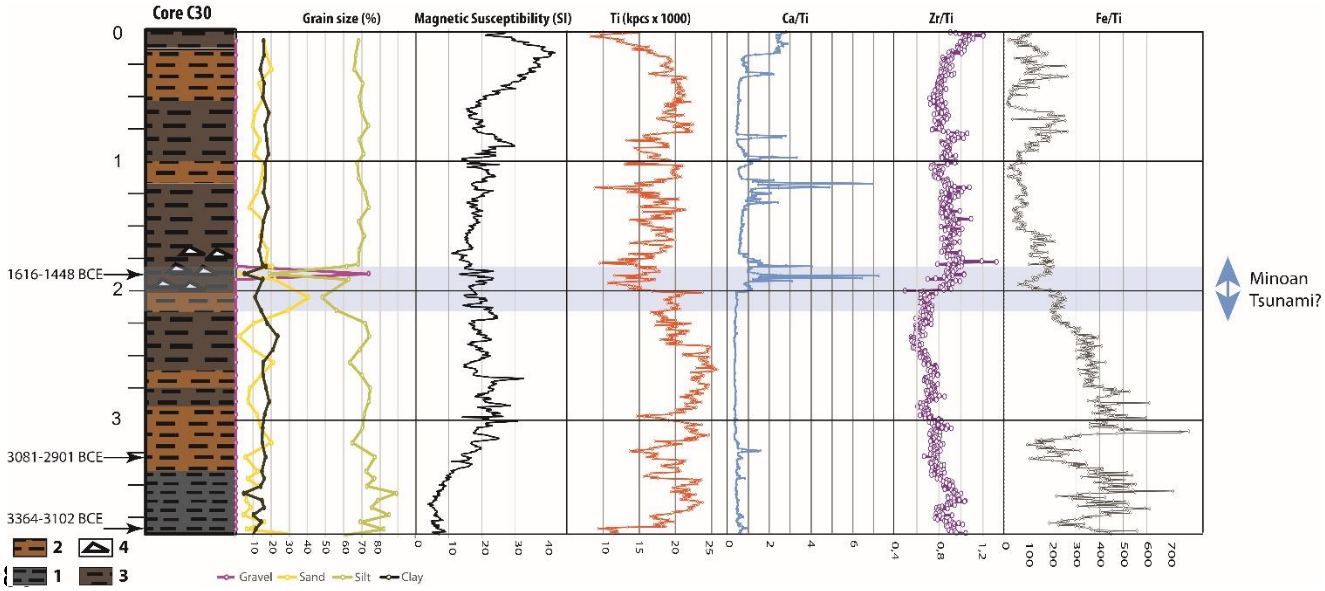

**Fig. S11. Grain-size distribution of Sand beach sampled 30cm under the current beach surface**

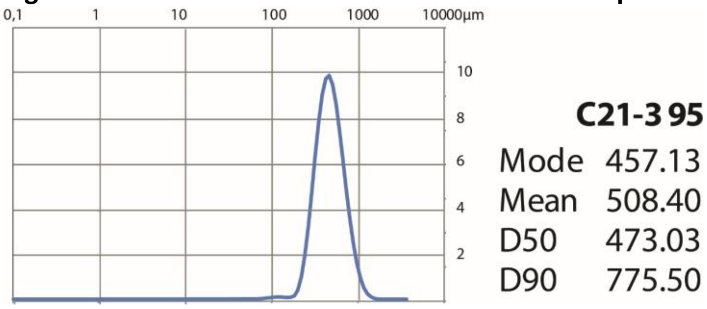

**Fig. S12. Microfaunal analyses of samples from cores C20, C22, C26, C29, C30**

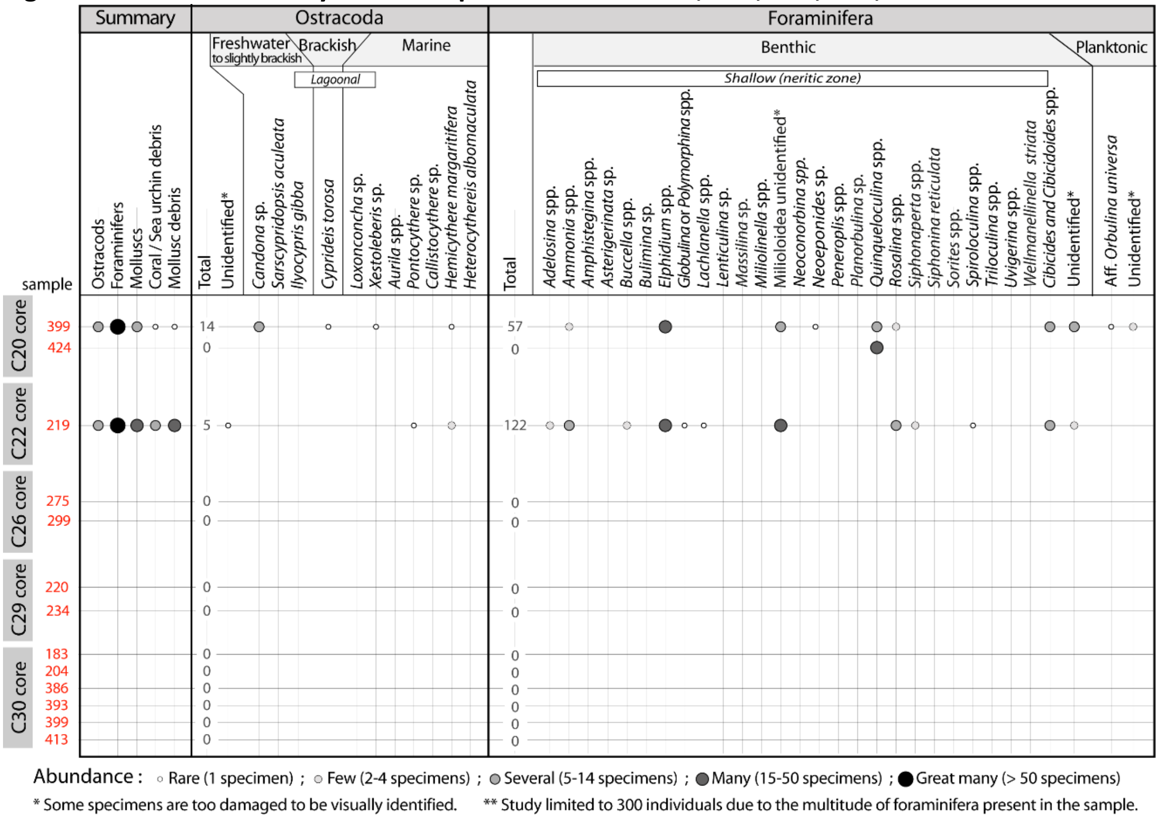

**Fig. S13. Microphotographs of ostracods species identified in the samples of Malia cores C21, C20 & C22.** 1. Aurila convexa, LV, external view; 2. Aurila prasina, LV, external view; 3. Aurila sp., LV, external view; 4. Bairdia sp., RV, external view; 5. Aff. Callistocythere sp., LV, external view. 6. Candona aff. angulata, LV, external view; 7. Candona sp., RV, internal view; 8. Candona sp., LV, external view; 9. Cyprideis torosa, LV, internal view; 10. Cyprideis torosa, LV, external view. 11. Cyprinotus salinus, RV, external view; 12. Hemicythere margaritifera, RV, internal view; 13. Hemicythere margaritifera, LV, external view; 14. Heterocythereis albomaculata, LV, external view; 15. Ilyocypris gibba, RV, internal view. 16. Ilyocypris gibba, RV, external view; 17. Loxoconcha sp., LV, external view.

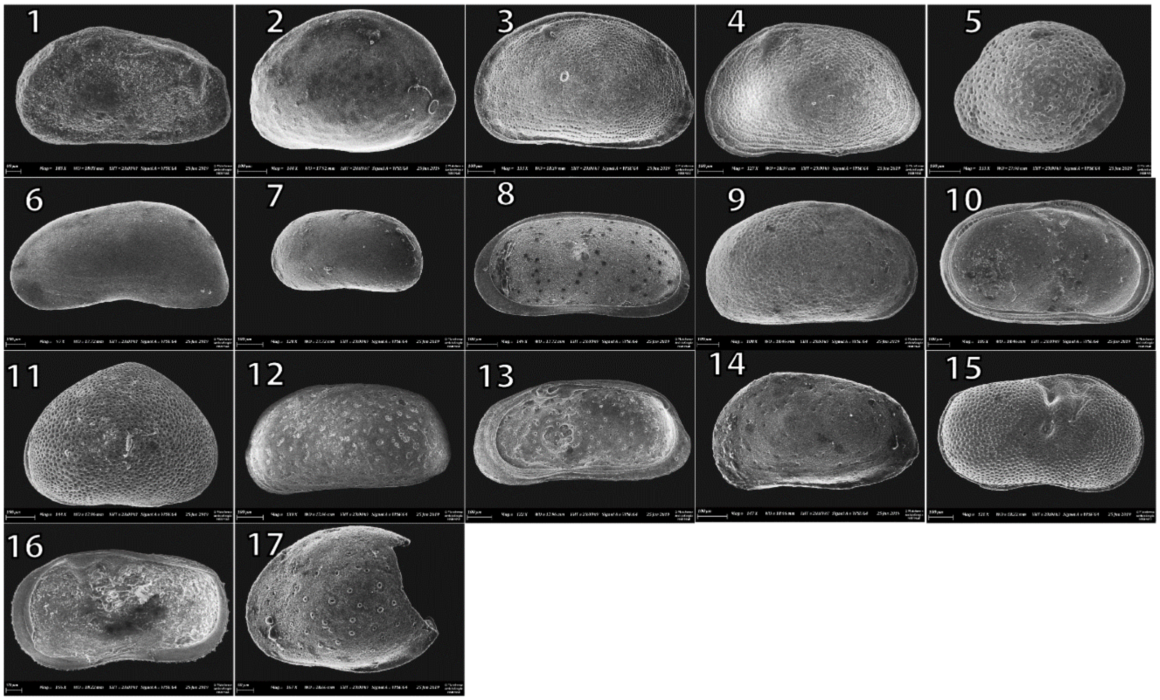

**Fig. S14. Microphotographs of benthic foraminifera species (more than 4 individuals) for sample C21-U4c-299.** 1. Ammonia spp.; 2. Elphidium spp.; 3. Cibicidoides lobatulus; 4. Elphidium crispum; 5. Quinqueloculina spp.; 6. Elphidium aff. Macellum; 7. Elphidium aff. Complanatum; 8. Cibicides refulgens; 9. Asterigerinata mamilla; 10. Buccella sp.; 11. Cibicidoides spp.; 12. Elphidium aff. Advenum; 13. Neoconorbina spp.; 14. Quinqueloculina aff. Stalkeri; 15. Elphidium aculeatum; 16. Rosalina spp.

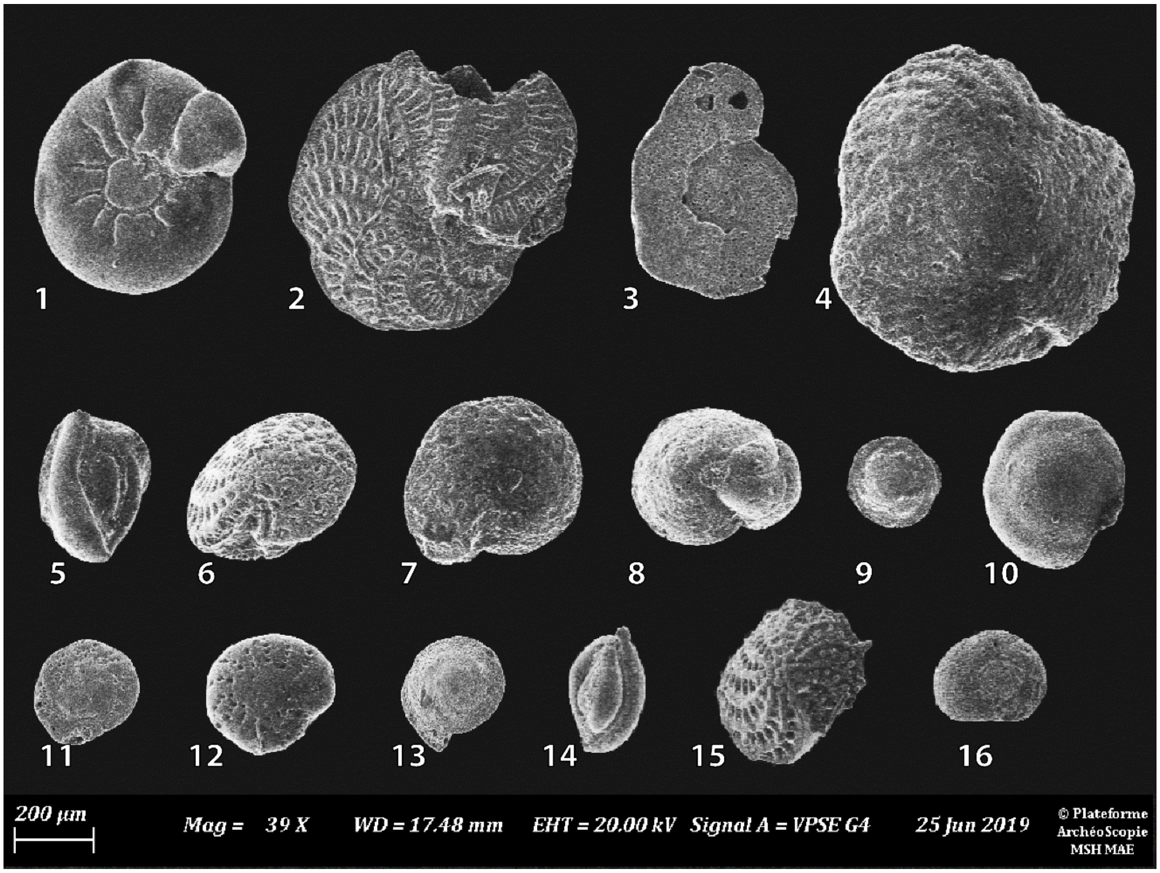

**Fig. S15. Microphotographs of planktonic foraminifera species identified in sample C21-U4c-299.** 1. Aff. Globorotalia menardii; 2. Aff. Neogloboquadrina sp.; 3. Aff. Neogloboquadrina sp.; 4. Aff. Orbulina sp; 5 to 15. Undetermined Globigerinitoidea or Globorotalioidea

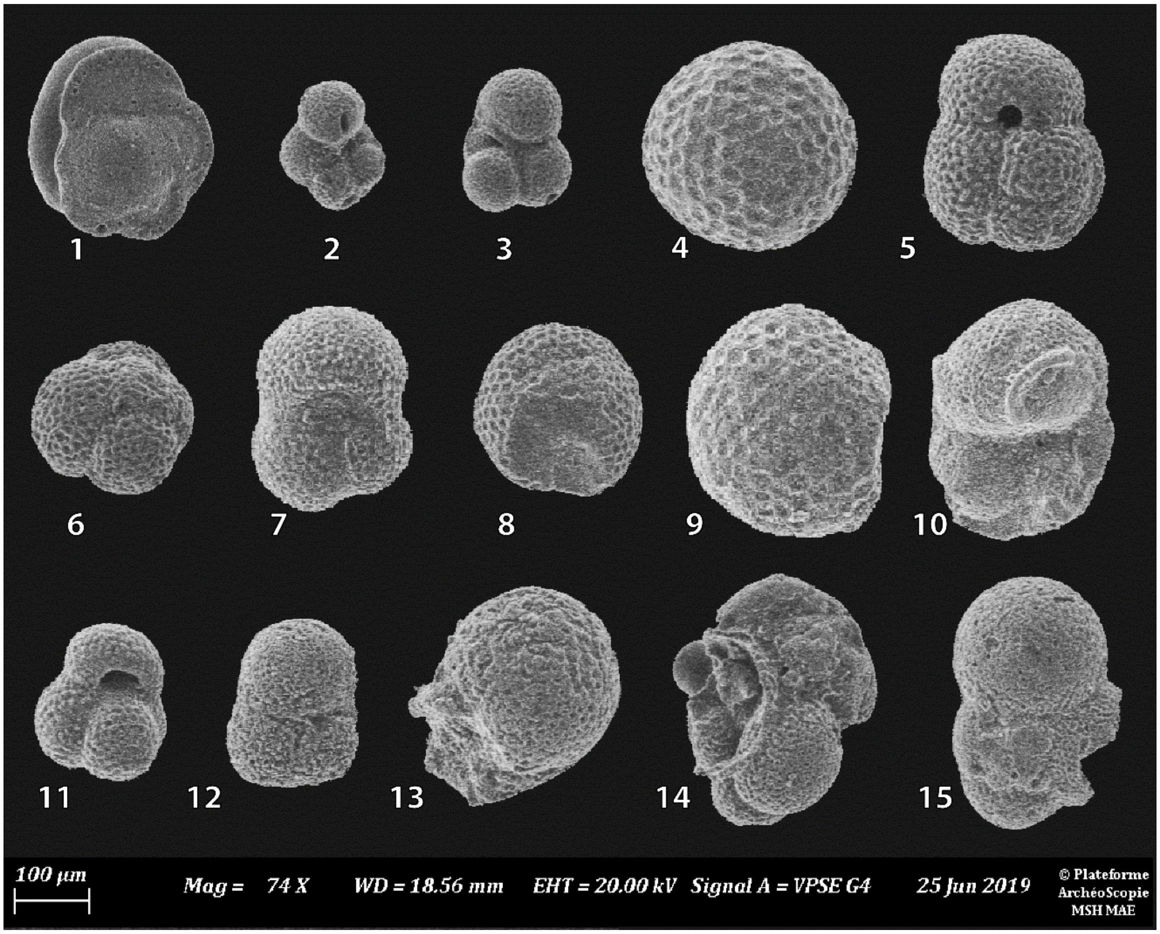

**Fig. S16. Microphotographs of benthic foraminifera species (more than 2 individuals) and Cibicides aff. Kullenbergi of sample C21-U4b-320.** 1. Cibicidoides lobatulus; 2. Ammonia sp.; 3. Elphidium crispum; 4. Elphidium aff. Complanatum; 5. Elphidium aff. Macellum; 6. Elphidium sp.; 7. Asterigerinata aff. Mamilla; 8. Rosalina aff. Bradyi; 9. Elphidium aff. Fichtelianum; 10. Cibicides sp.; 11. Rosalina aff. Obtuse; 12. Rosalina sp.; 13. Elphidium aff. Margaritaceum; 14. Cibicides aff. Kullenbergi.

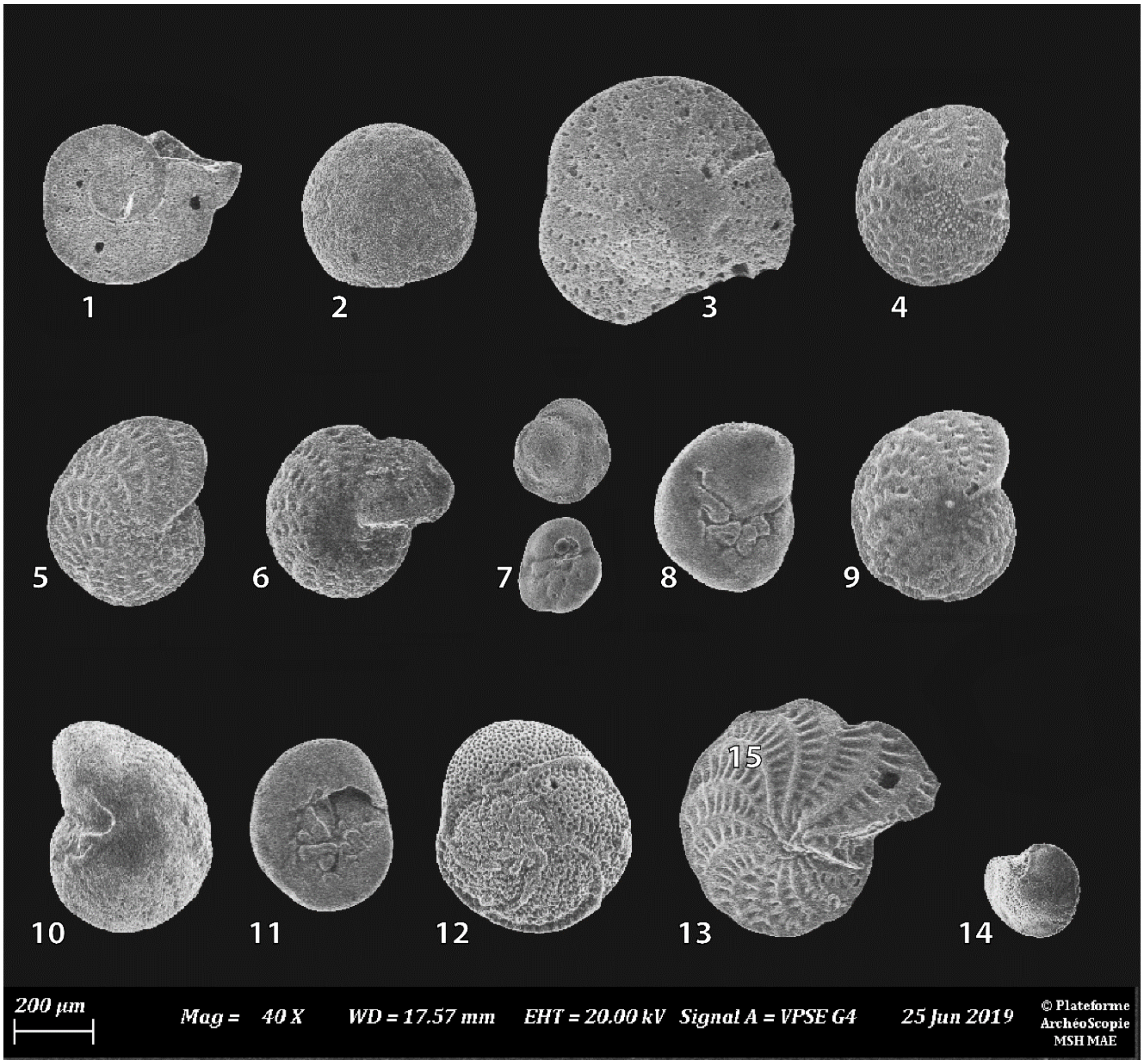

**Table S1. Table of the radiocarbon dates obtained from the Malia marsh sedimentary archives** *(in italic, rejected dating)*

| Core | Depth (m) | Material | $^{14}$C Age | Error 2σ | Cal. 2σ | Sample Code | Reference |
|---|---|---|---|---|---|---|---|
| **C6** | 2.00 | Gyttja | 536 | 35 | 1282-1490 CE | Lyon-7112 | Lespez et al., 2003 |
| ***C6*** | *2.18* | *Gyttja* | *1910* | *70* | *47 BCE-317 CE* | *Lyon-7113* | Lespez et al., 2003 |
| **C6** | 2.73 | Gyttja | 1770 | 55 | 133-413 CE | Lyon-7114 | Lespez et al., 2003 |
| **C6** | 2.97 | Gyttja | 2020 | 70 | 337 BCE-203 CE | Lyon-7115 | Lespez et al., 2003 |
| **C6** | 3.755 | Gyttja | 3340 | 50 | 1743-1505 BCE | Lyon-7116 | Lespez et al., 2003 |
| **C6** | 4.04 | Plant remain | 3790 | 45 | 2448-2040 BCE | Lyon-7117 | Lespez et al., 2003 |
| **C6** | 4.175 | Charcoal | 3955 | 50 | 2578-2293 BCE | Lyon-7118 | Lespez et al., 2003 |
| **C6** | 4.83 | Gyttja | 5080 | 65 | 4037-3662 BCE | Lyon-7119 | Lespez et al., 2003 |
| **C6** | 4.99 | Gyttja | 5210 | 55 | 4235-3819 BCE | Lyon-7120 | *Lespez et al., 2003* |
| ***C6*** | *5.32* | *Plant remain* | *4765* | *165* | *3950-3094 BCE* | *Lyon-7121* | Lespez et al., 2003 |
| **C6** | 7.03 | Gyttja | 7440 | 80 | 6439-6088 BCE | Lyon-7122 | Lespez et al., 2003 |
| **C28** | 1.11 | Charcoal | 1040 | 30 | 893-1114 CE | Poz-85400 | This study |
| ***C28*** | *1.3* | *Charcoal* | *100.3* | *0.35pMC* | *Modern* | *Poz-85401* | *This study* |
| **C28** | 3.00 | Charcoal | 3995 | 35 | 2623-2411 BCE | Poz-85441 | This study |
| ***C28*** | *3.31* | *Charcoal* | *3800* | *35* | *2403-2062 BCE* | *Poz-85756* | *This study* |
| **C28** | 3.8 | Charcoal | 4220 | 30 | 2905-2678 BCE | Beta-510919 | This study |
| **C28** | 4.16 | Gyttja | 4840 | 35 | 3702-3527 BCE | Poz-85757 | This study |
| **C28** | 4.31 | Gyttja | 5815 | 35 | 4782-4550 BCE | Poz-85759 | This study |
| **C26** | 1.255 | Gyttja | 585 | 30 | 1303-1416 CE | Poz-83103 | This study |
| **C26** | 1.875 | Gyttja | 1370 | 30 | 605-772 CE | Poz-83102 | This study |
| **C26** | 2.33 | Gyttja | 2015 | 30 | 95 BCE-109 CE | Poz-83104 | This study |
| **C26** | 2.825 | Gyttja | 3270 | 30 | 1616-1456 BCE | Poz-83106 | This study |
| **C26** | 3.275 | Wood | 3500 | 30 | 1919-1701 BCE | Poz-83107 | This study |
| **C26** | 3.94 | Gyttja | 4110 | 30 | 2867-2573 BCE | Poz-83108 | This study |
| **C26** | 4.295 | Gyttja | 5155 | 30 | 4045-3811 BCE | Poz-83109 | This study |
| **C26** | 4.825 | Gyttja | 5340 | 40 | 4324-4049 BCE | Poz-83110 | This study |
| **C26** | 5.435 | Gyttja | 5930 | 40 | 4932-4714 BCE | Poz-83111 | This study |
| **C26** | 5.715 | Gyttja | 6230 | 40 | 5306-5054 BCE | Poz-83112 | This study |
| **C30** | 1.94 | Charcoal | 3255 | 35 | 1613-1446 BCE | Poz-85336 | This study |
| ***C30*** | *2.855* | *Charcoal* | *2825* | *30* | *1107-901 BCE* | *Poz-85338* | *This study* |
| **C30** | 3.00 | Charcoal | 4520 | 30 | 3360-3101 BCE | Beta-480127 | This study |
| **C30** | 3.28 | Charcoal | 4350 | 30 | 3077-2899 BCE | Poz-85339 | This study |
| **C30** | 3.88 | Charcoal | 4535 | 35 | 3366-3101 BCE | Poz-85340 | This study |
| **C30** | 4.41 | Charcoal | 4500 | 35 | 3357-3041 BCE | Poz-85341 | This study |
| **C30** | 4.92 | Charcoal | 4480 | 30 | 3341-3031 BCE | Beta-451310 | This study |
| ***C29*** | *2.985* | *Plant remain* | *4920* | *30* | *3769-3642 BCE* | *Beta-451311* | *This study* |
| **C29** | 3.62 | Gyttja | 4690 | 30 | 3605-3370 BCE | Beta-451312 | This study |

| | | | | | | | |
|---|---|---|---|---|---|---|---|
| **C29** | 4.95 | Charcoal | 4720 | 30 | 3628-3375 BCE | Beta-451313 | This study |
| **C22** | 0.7 | Charcoal | 2000 | 80 | 197 BCE-218 CE | Poz-96172 | This study |
| **C22** | 1.24 | Charcoal | 3115 | 35 | 1492-1279 BCE | Poz-96173 | This study |
| **C22** | 2.11 | Charcoal | 3200 | 30 | 1513-1417 BCE | Poz-96175 | This study |
| **C20** | 2.67 | Charcoal | 3220 | 30 | 1533-1427 BCE | Poz-85442 | This study |
| **C21** | 1.16 | Charcoal | 210 | 420 | Post 979 CE | Poz-96170 | This study |
| **C21** | 1.96 | Charcoal | 820 | 35 | 1168-1274 CE | Poz-96171 | This study |
| ***C21*** | *2.86* | *Gyttja* | *4070* | *30* | *2850-2488 BCE* | *Poz-95029* | This study |
| **C21** | 2.895 | Gyttja | 2585 | 30 | 811-592 BCE | Poz-105990 | This study |
| **C21** | 2.995 | Charcoal | 3490 | 35 | 1920-1694 BCE | Poz-105975 | This study |
| **C21** | 3.22 | Charcoal | 3425 | 35 | 1876-1622 BCE | Poz-105979 | This study |
| **C21** | 3.44 | Gyttja | 3380 | 35 | 1751-1540 BCE | Poz-95778 | This study |
| **C21** | 3.67 | Gyttja | 3800 | 35 | 2403-2062 BCE | Poz-95779 | This study |

**Table S2. Table of the radiocarbon dates obtained from peat layer under the tephra deposits at Gölhisar lake**

| Site | Core | Depth (m) | Material | $^{14}C$ Age | Error 2σ | Cal. 2σ | Sample Code | Reference |
|---|---|---|---|---|---|---|---|---|
| Gölhisar (Turkey) | GHA.92-3 | 0.59-0.63 | Peat | 3300 | 70 | 1674-1499 | Beta-56673 | Eastwood et al. 1999 |
| Gölhisar (Turkey) | GHE-93-7 | 0.13-0.21 | Peat | 3225 | 45 | 1528-1441 | SRR-5188 | Eastwood et al. 1999 |

**SI References**


S1. Raban, A., Minoan and Canaanite harbours.Thalassa: l'Egée préhistorique et la mer, in: *Actes de la troisième Rencontre égéenne internationale de l'Université de Liège* (Aegaeum **7** 1991), pp. 129-146.

S2. Reimer, P. J., McCormac, F. G., Marine radiocarbon reservoir corrections for the Mediterranean and Aegean Seas. *Radiocarbon* **44** (1), 159-166 (2002)..

S3. Boaretto, E., Mienis, H. K., Sivan, D., Reservoir age based on pre-bomb shells from the intertidal zone along the coast of Israel. *Nuclear Instruments and Methods in Physics Research B* **268**, 966-968 (2010).

S4. Blaauw, M., and Christen, J. A. (2011), "Flexible paleoclimate age-depth models using an autoregressive gamma process," Bayesian Analysis, 6(3), 457–474.
